# Linking microbiome structure to functional analysis identifies resilient *Pseudarthrobacter*, *Pseudomonas*, and *Streptomyces* antagonists of *Phytophthora infestans* in tomato

**DOI:** 10.3389/fmicb.2026.1810932

**Published:** 2026-05-01

**Authors:** Philemon Orwa, Theresa Kuhl-Nagel, Rosa Meinhold-Ernst, Johannes A. Jehle, Romano Mwirichia, Ada Linkies

**Affiliations:** 1 Julius Kühn Institute (JKI) - Federal Research Centre for Cultivated Plants, Institute for Biological Control, Dossenheim, Germany; 2 Leibniz Institute of Vegetable and Ornamental Crops (IGZ), Plant-Microbe Systems, Großbeeren, Germany; 3 Department of Biological Sciences, University of Embu, Embu, Kenya

**Keywords:** 16S rRNA, Alternaria solani, bacterial microbiome, biological control, Illumina sequencing, microbial antagonists, *Pseudomonas syringae* pv. tomato, *Xanthomonas* vesicatoria

## Abstract

**Introduction:**

Late blight, caused by *Phytophthora infestans*, remains one of the most destructive tomato diseases, driving the need for sustainable measures to reduce intensive fungicide use. Plant-associated microbial communities offer a promising but still underexplored source of biological control agents. Moreover, links between community-level microbiome patterns and functionally effective antagonists remain poorly resolved.

**Methods:**

In this study, we combine culture-dependent isolation, functional profiling of lytic enzymes and siderophores, with 16S rRNA Illumina-based microbiome analysis to identify ecologically relevant bacterial antagonists of *P. infestans* in tomato. Healthy and *P. infestans*-challenged tomato plants cultivated in soils from two organic tomato farms in the Rhine-Main region of Germany were analyzed.

**Results:**

Of the 594 bacterial isolates from tomato rhizosphere and phyllosphere, 84 inhibited *P. infestans*, and 63 of these suppressed *Alternaria solani in vitro*. Functional screening identified 28 isolates with broad-spectrum antagonistic potential, predominantly affiliated with the genera *Pseudomonas, Bacillus, Streptomyces, Paenibacillus*, and *Pseudarthrobacter*, characterized by broad siderophore and taxon-specific lytic enzyme activities. *In planta* assays identified *Pseudarthrobacter* sp._Pb177 as a novel and most effective antagonist of *P. infestans*, alongside effective *Streptomyces* and *Pseudomonas* isolates. Amplicon-based microbiome analyses of different tomato compartments under both healthy and *P. infestans*-challenged conditions revealed soil origin as the primary driver of bacterial community assembly, particularly in the rhizosphere and phyllosphere. Disease-associated shifts were limited to specific soil-compartment combinations (rhizosphere soil B). Key rhizosphere bacterial taxa (*Acinetobacter* and *Chryseobacterium*) remained largely stable across plant health states. Instead, disease effects are confined to shifts among rare or conditionally detected ASVs. Mapping cultured isolates to amplicon sequence variants demonstrated that most antagonists corresponded to low-abundance members of the tomato microbiome (including *Bacillus, Chryseobacterium, Paenibacillus, Pseudomonas, Streptomyces*, etc.), with their distribution shaped primarily by soil and plant compartment rather than disease.

**Discussion:**

These findings indicate that effective biocontrol candidates are defined less by abundance than by their resilience and function within plant-associated microbial communities. By linking microbial community profiling with functional screening and *in planta* assays, this study outlines a microbiome-informed approach for identifying bacterial antagonists of *P. infestans* and supports an ecologically grounded framework for managing tomato late blight.

## 1 Introduction

Tomato (*Solanum lycopersicum*) is a staple crop cultivated in about 3.7 million hectares worldwide and is valued for its nutrition and culinary versatility (Lamsal et al., 2013). Tomato production faces serious threats from various plant pathogens, among them *Phytophthora infestans.* As the causative agent of late blight, this oomycete ranks among the most destructive and persistent plant pathogens, in particular on potato and tomato plants (Hashemi et al., 2022; Wang et al., 2023). Late blight continues to impose a major economic burden on global tomato cultivation, through severe yield reductions, high reliance on fungicide applications, and associated post-harvest losses across affected crops (Esquivel-cervantes et al., 2022; Liu et al., 2018). Given the fundamental role of tomatoes in global human nutrition, the continuous threat posed by *P. infestans* remains a persistent challenge for food security. The life cycle of *P. infestans* involves rapid and abundant spore production, a short infection cycle under optimal conditions, and effective dispersal, even active distribution of motile zoospores, which complicates effective disease management (Fan et al., 2021; Leesutthiphonchai et al., 2018). The pathogen rapidly evolves due to its highly compartmentalized genome, where infection-related and adaptive genes are concentrated in repetitive regions prone to accelerated evolutionary change (Mandal et al., 2022). Traditional management methods, among them application of chemical fungicides and breeding for host resistance, face major limitations, primarily because the pathogen can rapidly develop fungicide resistance and adapt to new host resistance genes (Haas et al., 2009; Khadka et al., 2020; Raffaele et al., 2010).

Plant disease management has traditionally relied on host genetic resistance, cultural practices, and the use of synthetic pesticides (Shattock, 2002; Tran et al., 2007). Chemical control can disrupt microbial community balance, reducing the activity of beneficial microbes and, at the same time promote the emergence of pesticide-resistant pathogen strains (Shanmugam and Kanoujia, 2011). Copper can harm humans, animals, and the environment, driving the need to identify and implement more efficient and eco-friendly control strategies.

An environmentally sound alternative to chemical control is the use of microbial antagonists (Kang et al., 2021; Koch et al., 2018). Biological control products currently account for only a small share of the global crop protection market (USD 5 billion), but are projected to triple in value, nearly USD 15 billion by 2029, driven largely by microbial biopesticides (Marrone, 2024). Biological agents derived from living organisms are known to reduce plant pathogen populations by direct antagonism, such as growth suppression, metabolite secretion, or by indirect effects, for example, the induction of plant defense reactions (O’Brien, 2017).

In the past decade, antagonistic activity against *P. infestans* has been described for both fungal and bacterial microorganisms, with each group offering distinct advantages depending on the ecological context and application strategy (Hashemi et al., 2022; Leesutthiphonchai et al., 2018; Wang and Long, 2023). Among bacterial antagonists, members of the genera *Bacillus, Pseudomonas*, and *Streptomyces* have been described frequently (Alfiky et al., 2022; Caulier et al., 2018; Daayf et al., 2003; Kloepper et al., 2004; Mendes et al., 2011). Beyond their widespread occurrence and strong colonization capacity across diverse environments, several beneficial bacteria produce robust antimicrobial metabolites that show considerable potential as alternatives to synthetic fungicides (Anand et al., 2020; Caulier et al., 2018; Wang et al., 2020). However, successful biocontrol requires antagonists that are not only effective *in vitro* but also ecologically adapted to the plant microbiome.

Plant-associated microbiomes are spatially structured into discrete habitats, the rhizosphere, phyllosphere, and endosphere, with each compartment supporting a characteristic microbial community shaped by plant genotype, plant organs, and environmental conditions (Berg et al., 2016; Dong et al., 2019; Zhang et al., 2020). The phyllosphere supports lower but specialized microbial diversity shaped by environmental stress (Vorholt, 2012), whereas the endosphere hosts distinct endophytic communities that contribute to plant growth and disease tolerance (Hardoim et al., 2015). The rhizosphere is a highly active microbial hotspot enriched with the most diverse plant-associated bacterial communities, strengthening plant systemic resistance, promoting plant growth, and harboring antagonistic taxa, which can suppress plant pathogens (Berendsen et al., 2012; Berihu et al., 2023; Mendes et al., 2013; Vinothini et al., 2024). Disease suppression in plants is increasingly recognized as a community-level trait driven by microbiome diversity and functional composition rather than by individual antagonists (Ehau-Taumaunu et al., 2025; Hu et al., 2016; Tao et al., 2020). Accordingly, disease-suppressive soils are characterized by distinct, functionally rich microbial communities in the rhizosphere (Shan et al., 2024).

Despite substantial progress in microbiome research and biocontrol screening, few studies directly link microbial community composition with functional antagonism against major pathogens such as *P. infestans*. Most studies either focus on profiling the microbiome or on isolating and testing antagonists (Ehau-Taumaunu et al., 2025; Hashemi et al., 2022; Zhou et al., 2025), but rarely integrate these approaches to link ecological relevance with biocontrol function. In addition, large-scale *in vitro* screening has identified many potential bacterial biocontrol agents against *P. infestans* (Hashemi et al., 2022), yet their ecological relevance within the native plant microbiome remains largely unexplored.

In this study, we combined isolation of culturable bacteria from healthy and *P. infestans* infected tomato plant tissues with 16S rRNA amplicon sequencing of the same tissues, functional screening, and amplicon sequence variants (ASV)-isolate mapping to: (i) identify bacterial antagonists of *P. infestans* and characterize their lytic enzyme and siderophore activity profiles, (ii) assess their inhibitory potential *in vitro* and *in planta*, (iii) evaluate whether pathogen infection alters the structure of the tomato-associated bacterial microbiome, and (iv) determine whether selected antagonists represent dominant and ecologically relevant members of the tomato-associated microbiome across contrasting soil origins. Collectively, this integrative approach sought to link microbiome structure with functional biocontrol to advance a microbiome-informed strategy for the sustainable management of tomato late blight.

## 2 Materials and methods

### 2.1 Experimental design of the tomato-late blight-pathosystem

The experimental design follows procedures described in detail in our previous work Orwa et al. (2025). Soil used for tomato cultivation in this experiment was collected from greenhouse tomato production sites of two organic farms in the German Rhine-Main area: Domäne Mechthildshausen, Wiesbaden, 50°02′ N, 8°19′ E (soil A) and Solidarische Landwirtschaft Rüsselsheim, 49°58′ N, 8°25′ E (soil B). Soil samples were transferred to the greenhouse at the Julius Kühn Institute (JKI), Institute for Biological Control, Dossenheim, Germany, in March 2023, for further experimentation.

Briefly, from each site, soil was sampled at five evenly distributed positions within the greenhouse at 20 cm depth, pooled and homogenized to obtain one composite substrate per location. Soils A and B were transported separately to JKI, Dossenheim, Germany, and used to grow tomato plants (cv. ‘Red Robin’) under controlled greenhouse conditions. Fourteen-day-old seedlings were transplanted to individual pots (8 × 8 × 8.5 cm) and grown for three additional weeks before four plants per soil were inoculated with 7 mL of sporangia suspension (2 × 10^4^ sporangia/mL) of *Phytophthora infestans* (isolate 606, JKI culture collection) onto the leaves. Four plants from each soil origin served as healthy controls. Plants were placed in a climate chamber (temperature: 21°C; humidity 75%; 12/8 h light regime; light 100 lux) and disease severity was assessed 2 weeks post-inoculation as the percentage of symptomatic leaf area. Healthy control plants did not show any symptoms. Leaves, roots, and rhizosphere soil from the diseased and healthy plants were used in the subsequent experiments for the isolation of bacteria and for microbiome studies.

### 2.2 Sample preparation for bacterial DNA extraction and isolation of viable bacteria

Plant material from the phyllosphere (leaves), endosphere (roots), and rhizosphere soil of diseased and healthy tomato plants was prepared separately for 16S rRNA gene amplicon sequencing and for isolation of viable bacteria as described comprehensively in Orwa et al. (2025). For microbiome profiling, each plant was treated as an independent biological replicate (*n* = four plants per soil origin × plant condition). Sample processing followed Wieland et al. (2001) and Semagn (2014), with slight modifications.

To prepare the rhizosphere sample, roots were shaken to remove loose soil. Approximately 5 g of adhering soil was vortexed in saline buffer (0.85% NaCl) for 2 min. Roots (approximately 5 g) were then vortexed for 5 min in saline buffer to release the rhizoplane, which was then combined with the rhizoplane suspension. To obtain an endosphere sample, roots (100–150 mg) were surface-sterilized following a series of 70% ethanol and 50% sodium hypochlorite, followed by five rinses in sterile distilled water. The cleaned roots were then homogenized in sterile saline buffer. To obtain phyllosphere samples, material (100–150 mg) was harvested by pooling leaves from four branches. For diseased plants, asymptomatic leaf tissue adjacent to lesions was collected.

### 2.3 Isolation of cultivable bacteria from tomato tissues

To establish a broad working collection for functional screening, bacteria were isolated from above-ground (phyllosphere) and below-ground (root-associated) compartments of healthy and diseased plants grown in the two soils. Pooling was applied only for cultivation-based isolation: within each soil origin (A and B) × plant condition group, material from the four replicate plants was combined to maximize recovery of culturable diversity, resulting in four composite isolation sources per compartment (two soils, A and B) × (two plant conditions). In addition, for the purpose of isolate recovery, endosphere and rhizosphere fractions were combined to represent a single below-ground inoculum (“root-associated/rhizosphere source”), whereas phyllosphere samples represented the above-ground source. Each sample was serially diluted (10^3^–10^6^) in sterile saline solution (0.85% NaCl), and 100 μL of each dilution was spread-plated onto two culture media: nutrient agar (NA) and M9 minimal agar (MA) supplemented with chloramphenicol (20 mg/mL). The plates were incubated at 14, 21, and 28°C to broaden the spectrum of recoverable bacterial taxa. Colony development was first examined after 3 days of incubation, and morphologically distinct colonies depicting diversity across media, temperatures, and sample types were selected. These colonies were subsequently purified through repeated streaking, resulting in a representative collection of 594 cultivable bacteria after around 7 days of incubation (Supplementary Excel File 1). Because isolates were obtained from pooled material, isolation outputs were used to build a representative collection and for downstream functional assays rather than for statistical inference of abundance differences among treatments.

### 2.4 Stepwise screening and identification of bacterial antagonists against *P. infestans* and other tomato pathogens

To identify bacterial candidates for biocontrol, the methodology followed the stepwise screening framework established by Köhl et al. (2011), which allows for the selection of bacteria that fulfill requirements for commercial use other than antagonistic efficacy, including functional and ecological characteristics, growth parameters, and cost-effective production.

#### 2.4.1 Dual-culture screening for inhibitory potential of bacterial antagonists against fungal and bacterial plant pathogens

A total of 594 bacterial isolates were screened for inhibitory potential against *P. infestans* and *Alternaria solani* (isolate 62028 of JKI culture collection) *in vitro* using dual-culture assays (Supplementary Excel File 1). Briefly, a mycelial plug (0.8 cm) of *P. infestans* culture (14-day-old, grown in rye agar at 15°C) and *A. solani* culture (14-day-old, grown on V8 medium at 21°C) was placed individually at the center of a Petri dish with nutrient agar. Subsequently, 10 μl of a 24-h-old bacterial culture, grown in nutrient agar in 50 mL Falcon tubes at 26°C, was spot-inoculated 4 cm apart from the fungal mycelial plug. The control had only the mycelial plugs from the fungal pathogens at the center of the Petri dish. The Petri dishes were incubated at 21°C for 5 days for assays with *P. infestans* and for 14 days for assays with *A. solani*. Antagonistic activity was determined by the presence or absence of a clear inhibition zone forming between the bacterial colony and the fungal plug. Results were scored as “yes” (zone present) or “no” (zone absent).

A total of 116 bacterial isolates inhibiting both *P. infestans* and *A. solani* or *P. infestans* exclusively *in vitro* were selected for subsequent screening. Of these, only 84 bacteria were retained after eliminating candidates that lost both their antagonistic effects and viability within repeated screenings (Supplementary Excel File 1). The 84 identified antagonistic bacteria were screened for their suppressive activity against *P. infestans* on rye agar (200 g rye; 3 g glucose; 12 g agar; 1,000 mL distilled water) in dual culture assays as described above. This screening was performed once on three replicate Petri dishes.

To evaluate broad-spectrum antagonistic effects, these candidate antagonists were further tested for their inhibitory effects on tomato-related bacterial pathogens, namely *Pseudomonas syringae* pv. *tomato* and *Xanthomonas vesicatoria* using the cross streak method (Balouiri et al., 2016) (Supplementary Excel File 2). Briefly, a single primary streak from a 24–72 h-old antagonistic bacterial culture, grown in nutrient broth at 26°C, was made along the center of a nutrient agar plate. A secondary streak from a 24 h-old pathogenic bacterial culture (*P. syringae* pv. *tomato* (left side of the plate) and *X. versicatoria* (right side of the plate) were then made perpendicular to, but not touching, the streak. The plates were incubated at 25°C for up to 72 h. Antagonism was confirmed by the presence of a visible inhibition zone between the candidate streak and the target pathogen. Results were scored as “yes” (zone present) or “no” (zone absent). The *in vitro* confrontation assays with the two bacterial pathogens were used as the primary selection criteria at this stage to identify the most promising candidates; the bacterial pathogens were not included in subsequent experimental steps.

#### 2.4.2 PCR identification of bacterial isolates

The 84 candidate antagonists were identified up to the genus level by sequencing the 16S rRNA gene. Briefly, bacterial DNA was extracted from 24 to 72-h old cultures grown on nutrient broth at 25°C using the DNeasy Ultra Clean microbial kit (Qiagen, Germany) following the manufacturer’s instructions.

The V1-V3 region of the 16S rRNA gene was amplified by PCR using the primer pair 27F (5′- AGAGTTTGATCMTGGCTCAG-3′) and 511R (5′-GCGGCTGCTGGCACRKAGT-3′). The PCR amplification in a 25 μL reaction mix was prepared following the manufacturer’s specifications (Axon Labortechnik, Kaiserslautern). Unsuccessful amplifications were repeated using the GoTaq^®^ G2 Flexi DNA polymerase (Promega). The cycling parameters included an initial denaturation (95°C for 3 min), 40 cycles (95°C for 30 s; 54°C for 30 s; 72°C for 1 min, and a final extension (72°C for 5 min). PCR products were treated with ExoSAP-IT™ PCR Product Cleanup Reagent (Thermo Fisher Scientific), and 10 μL of the clean product was used for Sanger sequencing (StarSEQ). Sequence identification relied on Basic Local Alignment Search Tool (BLAST) analysis against the National Center for Biotechnology Information (NCBI) database blastn (Madden, 2013), with results prioritized by highest query coverage and percentage identity. Following the same procedure described above, the V1-V5 regions of the 16S rRNA gene from 28 bacterial isolates in the final collection were amplified by PCR using the primer pair 27F (5′- AGAGTTTGATCMTGGCTCAG-3′) and 907R (5′-CCGTCAATTCMTTTRAGTTT-3′) to obtain sequences overlapping with the 16S rRNA gene amplicon data from the microbiome analysis.

### 2.5 Selection of 28 bacterial antagonists for *in planta* trials

The 84 isolates above were further reduced to a manageable quantity to identify the most suitable antagonistic candidates for later plant trials using the following exclusion criteria: (i) literature review based on the preceding sequencing results to exclude bacterial genera that had been previously described as pathogenic to plants, human, or animals; (ii) literature evidence to include taxa previously reported potential as plant biocontrol agents; (iii) we considered strains that demonstrated broad spectrum inhibitory potential against *P. infestans* and the tested bacterial tomato pathogens, *P. syringae* pv. *tomato* and *X. vesicatoria*; (iv) reducing the number of strains from highly abundant genera (such as *Pseudomonas, Bacillus*, and *Streptomyces*). Our aim was to select strains representing the full taxonomic diversity of the isolate collection while minimizing redundancy from isolates sharing identical isolation conditions and plant origins. In the end, 28 bacterial strains were selected for initial plant tests. Concurrently, this collection was subjected to subsequent dual culture inhibitory tests against other tomato pathogens and tests for lytic enzyme activity *in vitro*. These functional screening was deemed useful for repeated plant trials and for consortia development in our future experiments.

#### 2.5.1 Dual-culture screening for inhibitory potential of bacterial antagonists against *R. solani, F. oxysporum*, and *Botrytis cinerea*

The 28 bacterial antagonists were subjected to a final round of *in vitro* screening, testing their inhibitory activity against three fungal pathogens of tomato: *Rhizoctonia solani*, *Fusarium oxysporum*, and *Botrytis cinerea*, grown on potato-dextrose agar (PDA) plates for 7 days and incubated at 25°C. The *in vitro* dual culture assays were performed as described above. All the 28 strains used in this comprehensive assessment yielded antagonistic candidates with broad-spectrum inhibitory effects, and were all considered for initial *in planta* trials on tomato plants against *P. infestans*.

#### 2.5.2 Activity of 28 bacterial antagonists for lytic enzymes and siderophores

**FIGURE 1:**
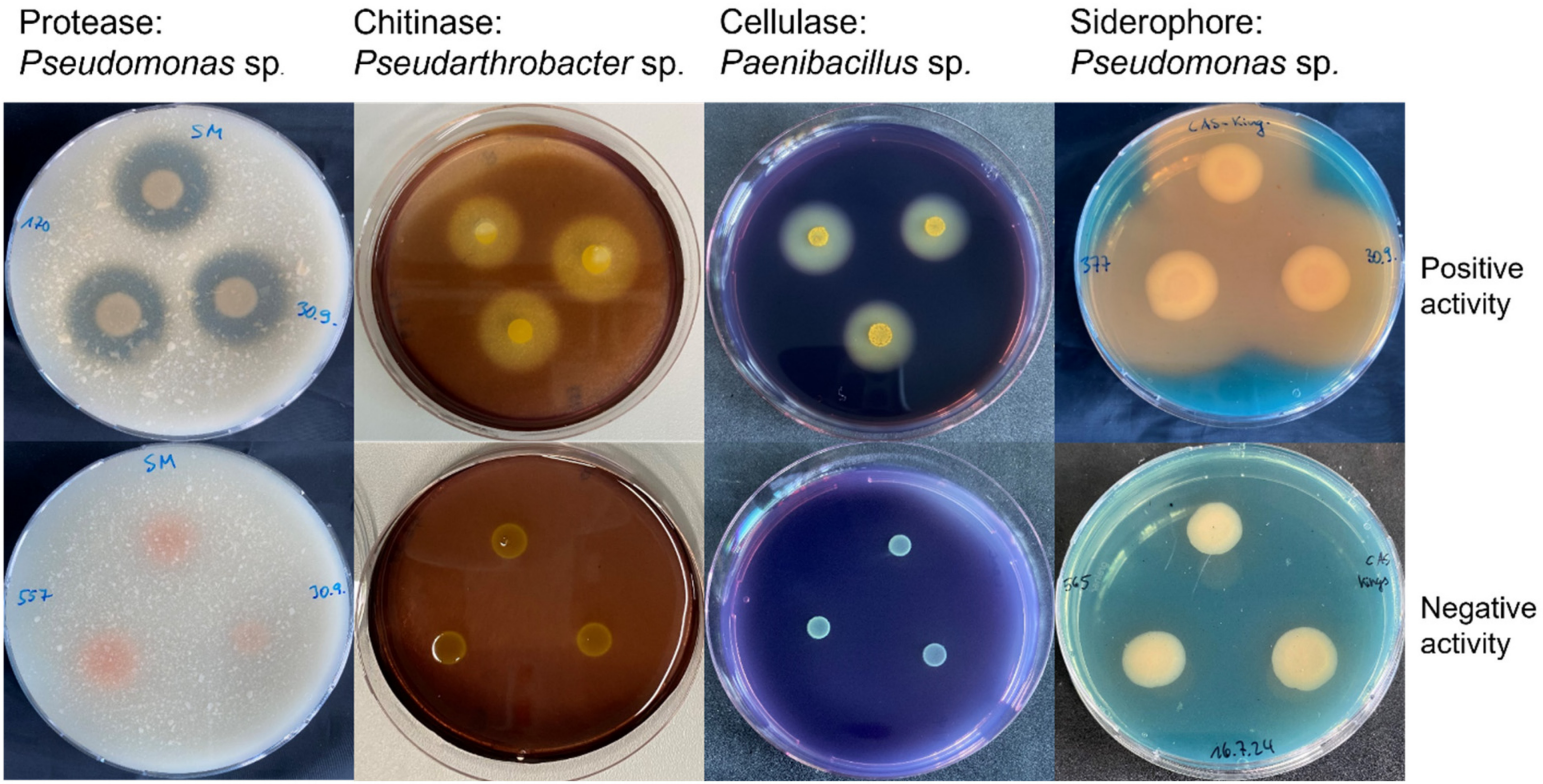
Lytic enzyme and siderophore activity plate assays. Halo formation, indicative of protease, cellulase, and chitinase activity, or siderophore production, is shown for representative bacterial antagonists **(upper row)** in comparison to the control with enzyme substrate in the medium **(bottom row)**. All assays were performed with three biological replicates and three technical replicates per treatment.

The production of lytic enzymes [cellulase (Ariffin et al., 2006), protease (Mounia et al., 2014), chitinase (Mounia et al., 2014), and siderophores (Neilands, 1981)] was assessed for the 28 selected bacterial candidates using the halo formation method on specific solid media. The presence of a clear halo surrounding the bacterial colony was indicative of activity (Figure 1). Each test was performed in triplicate (three biological replicates) with three technical replicates per run. At this stage, all the 28 strains used in this functional screening were all considered for initial *in planta* trials on tomato plants against *P. infestans*.

##### 2.5.2.1 Protease activity test

A 10 μL bacterial culture grown in nutrient broth (24–72 h, 26°C, 160 rpm) was spot inoculated onto skimmed milk (SM)-agar (skimmed milk, 28 g/L; casein hydrolysate, 5 g/L; yeast extract, 2 g/L; dextrose, 1 g/L; agar, 15 g/L at pH 7.0 ± 0.2). Plates were incubated for 24 h at 30°C and examined for zones of clearance.

##### 2.5.2.2 Chitinase activity test

A 10 μL bacterial culture in nutrient broth (24–72 h, 26°C, 160 rpm) was spot inoculated on chitin agar (NH_4_H_2_PO_4_, 1 g/L; KCl, 0.2 g/L; MgSO_4_.7H_2_O, 0.2 g/L; chitin from crab shells, 10 g/L; agar, 20 g/L at pH 6.0 ± 0.2). Plates were incubated for 120 h at 30°C and flooded with Gram’s iodine for 3–5 min to reveal zones of clearance.

##### 2.5.2.3 Cellulase activity test

A 10 μL bacterial culture grown in nutrient broth (24–72 h, 26°C, 160 rpm) was spot inoculated onto carboxy-methyl cellulose (CMC)-agar (KH_2_PO_4_, 1 g/L; MgSO_4_.7H_2_O, 0.5 g/L; NaCl, 0.5 g/L; FeSO_4_.7H_2_O, 0.01 g/L; MnSO_4_.H_2_O, 0.01 g/L; NH_4_NO_3_, 0.3 g/L; CMC, 10 g/L; agar, 12 g/L at pH 7.0 ± 0.2). Plates were incubated for 120 h at 30°C and flooded with Gram’s iodine for 3–5 min to reveal zones of clearance.

##### 2.5.2.4 Siderophore activity test

A 10 μL bacterial culture in nutrient broth (24–72 h, 26°C, 160 rpm) was spot inoculated on modified chrome Azurol S (CAS)-agar (10 mL of FeCl_3_.6H_2_O, 27 mg/100 mL of HCl 10 mM; 50 mL of CAS, 1.2 g/L; 40 mL of HDTMA, 1.82 g/L; 900 mL of LB-agar for other bacterial genera and King Broth agar for all *Pseudomonas* isolates at pH 6.8 ± 0.2). Plates were incubated for 120 h at 30°C and examined for zones of clearance.

### 2.6 Testing disease suppressive effects of selected bacterial isolates *in planta*

The disease-suppressive potential of 28 antagonistic bacterial isolates against *P. infestans* was evaluated in a two-stage *in planta* pipeline. First, an exploratory screening assay was conducted with a reduced number of plants (*n* = 3 per treatment) and a single experimental run. Aim was the down-scaling of candidates from a large pool to a manageable set for confirmatory testing. Second, a confirmatory trial was performed with nine bacterial candidates using six replicate plants per treatment and three independent experimental repetitions. The nine antagonists were selected based on their promising *in planta* efficacy in the first exploratory screening and their inhibitory effects against *Rhizoctonia solani*, *Fusarium oxysporum*, and *Botrytis cinerea in vitro*, and lytic anzyme activity profiles. The experimental setup was as follows: Tomato plants, cultivar “Red Robin” (Weigelt Samen, Germany) were grown by sowing seeds in a 3:1 mixture of commercial substrate (ProLine Potgrond, Klasmann-Deilmann, Germany) and sand in a greenhouse environment. Fourteen days post-sowing, the seedlings were individually transplanted into 8 × 8 × 8.5 cm planting pots, retaining the same soil composition. Tomato plants were irrigated demand-based and fertilized weekly with 0.2% (w/v) Hakaphos blau (Compo Expert, Germany). Bacterial antagonists were grown in nutrient broth for 72 h at 25°C in a shaker incubator at 160 rpm. Each of the above-mentioned bacterial strains was subsequently diluted to an OD (650nm) of 0.2 in distilled water. Five-week-old plants were sprayed on both sides of the leaves with approximately 5 mL of diluted bacteria and kept in darkness overnight. Tomato plants treated with Cuprozin progress (0.52%) and inoculated with *P. infestans* were used as the chemical control (copper standard). Plants inoculated with *P. infestans* without any treatment served as the diseased control, whereas water-treated, non-inoculated plants served as the healthy control. The following day, plants were spray-inoculated with 5 mL of *P. infestans* sporangia suspension (2 × 10^4^ sporangia/mL). To attain nearly 100% relative humidity required for infection, the trays containing the plants were covered with lids and kept in darkness overnight. The plants were moved to a climate chamber (Grow Bank, Plant Climatics, Germany) under 16 h light and 8 h darkness conditions. Further parameters were set as follows: day humidity at 65%, night humidity at 75%, day temperature at 21°C, and night temperature at 16°C. Disease severity was determined visually 7 days following leaf application with bacterial antagonists. The percentage of diseased leaf area was recorded according to the methodology established by Drenker et al. (2023). All plant trials, excluding the initial screening phase, adhered to a randomized design involving six tomato plants per treatment. The entire experimental workflow was conducted in three independent biological replicates.

### 2.7 DNA extraction and 16S rRNA gene sequencing

For 16S rRNA gene amplicon sequencing, total genomic DNA was extracted from 48 samples of the initial experiment using field soils (three compartments (rhizosphere, endosphere, phyllosphere) × four replicates × two soil origins (soil A and soil B) × two plant health conditions (diseased and healthy). For each sample, 0.25 mg material was processed using the DNeasy Powersoil Pro kit (Qiagen, Germany) according to the manufacturer’s instructions. Extracted DNA yield and purity were quantified photometrically using a NanoDrop 2000c (peqLab Biotechnologie GmbH, Germany). All DNA samples were stored at −20°C until further use. Library preparation and high-throughput amplicon sequencing of the V4 variable region of the 16S rRNA gene were commissioned to StarSEQ GmbH (Germany). The V4 region was amplified using the primer pair 515F (Caporaso et al., 2011) (5′ GTGCCAGCMGCCGCGGTAA-3′ and 799R (Artimová et al., 2022) (5′- CMGGGTATCTAATCCKGTT-3′). This primer set was selected as they exclude amplification of chloroplast sequences and minimizes the co-amplification of host mitochondrial DNA (Artimová et al., 2022). Illumina adapter sequences were incorporated into the amplicons in a 1-step PCR approach. The normalized libraries were sequenced on the Illumina MiSeq platform utilizing a V3 reagent kit, generating 2 × 300 nt paired-end reads.

### 2.8 Analysis of the 16S rRNA amplicon sequencing

Initial sequence quality control was performed using the software Cutadapt (v.5.2) (Martin, 2011) on the European Galaxy-Server ^1^ (Afgan et al., 2018), ensuring the removal of all primer and adapter sequences from the high-quality paired-end raw reads. The subsequent processing and merging of the paired reads were conducted in R (v.4.5.1) (R Core Team, 2022) using the DADA2 pipeline (v.1.36.0) (Callahan et al., 2016), following the established 16S pipeline workflow (1.8).^2^ From an initial pool of nearly 10,000,000 raw reads, the analysis yielded 6,733,088 quality-merged reads, with an average sequence depth of 140,273 ± 35,958 reads per sample (Supplementary Figure 1). The resulting amplicon sequence variants (ASVs) were taxonomically assigned using the Naive Bayesian Classifier (Wang et al., 2007) implemented in the assignTaxonomy function in the DADA2 R package, utilizing the SILVA nr99 database (version 138.1) (Quast et al., 2013) as the reference training set. Sequences detected as non-bacterial contaminants (mitochondria or chloroplasts) were filtered out. An additional filtering step removed amplicon sequence variants (ASVs) with an abundance of less than five reads, leaving a final, robust dataset containing 11,497 ASVs. Rarefaction curves confirmed that the sequencing depth achieved was sufficient for capturing the bacterial diversity in the samples (Supplementary Figure 1). All raw sequencing reads have been publicly deposited in the Sequence Read Archive (SRA)^3^ under the BioProject accession number PRJNA1273108. Furthermore, all scripts, including detailed parameters and the data necessary to replicate the figures in this work, are accessible on GitHub.^4^

### 2.9 Mapping 16S rRNA gene amplicon sequences with viable isolate 16S rRNA sequences

To compare our working collection of 28 bacterial species to the bacterial community in the microbiome, we mapped the ASV sequences (∼250 bp) to the isolate sequences (∼900 bp) with 100% identity and at least 50% query coverage using NCBI BLAST using the blastn tool in the European Galaxy-Server (see text footnote 1). The isolated strains were cross-referenced with the microbial community data to verify their inclusion within the highly abundant taxa and/or differentially abundant taxa. To link cultured isolates with microbiome profiles, ASVs showing 100% sequence identity to the isolate 16S rRNA gene sequences were identified and retained. For each linked isolate-to-ASV, rarefied read counts were converted to relative abundance (%) within each sample and averaged across experimental groups defined by microcompartment, soil origin, and plant health status. Mean relative abundance values were visualized using a heatmap generated with the ComplexHeatmap R package. Hierarchical clustering of isolates was performed using Bray-Curtis dissimilarity to account for the compositional structure of the data.

### 2.10 Data analysis, visualization, and statistics

All statistical processing and data manipulation for ASVs was conducted in R (v.4.5.1) (R Core Team, 2022) using the packages “ggplot2” (v.4.0.1) (Wickham, 2016), “tidyverse” (v.2.0.0) (Wickham et al., 2019), “tibble” (v.3.3.0) (Müller and Wickham, 2023), “ggtext” (v.0.1.2) (Wilke and Wienik, 2022), “dplyr” (v.1.1.4) (Wickham et al., 2023), “stringr” (v.1.6.0) (Wickham, 2019), “ARTool” (v.0.11.1) (Elkin et al., 2021), and “phyloseq” (v.1.52.0) (Paul and Susan, 2013), and “vegan” (v.2.7.2) (Oksanen et al., 2024).

#### 2.10.1 Analysis of 16S rRNA gene sequencing data

Microbiome analyses of the 16S rRNA gene were performed on plant-level replicates (*n* = 4). To control for uneven sequencing depth across samples, we rarefied read counts to the minimum library size of 14,886 reads using repeated subsampling (1,000 iterations). This strategy follows the framework advocated by Schloss (2024), who showed that rarefaction can provide robust control of uneven sequencing depth for alpha- and beta-diversity analyses. Rarefaction curves verified that this depth captured the majority of within-sample diversity (Supplementary Figure 1). Assessment of the influence of disease condition, soil origin, and micro-compartment on alpha diversity indices (richness, Shannon diversity index, evenness, Simpson index) was based on a three-way factorial analysis of variance (ANOVA) (Supplementary Tables 1–4). Non-parametric Aligned Rank Transform (ART) ANOVA was performed for strong deviations from normality according to the Shapiro-Wilk normality test (*p* ≤ 0.05); however, the *p*-values remained comparable to those of ANOVA. Beta diversity was assessed by first converting ASV counts to relative abundance (%), and then calculating Bray-Curtis dissimilarity matrices, which were subsequently visualized by non-metric multidimensional scaling (NMDS). The overall effects of disease status, soil, and microcompartment on the composition of the bacterial community were evaluated using PERMANOVA with 10,000 permutations (Supplementary Table 5). Initial visualizations of alpha and beta diversity indicated that the disease status effect was significant exclusively within the rhizosphere. Therefore, all detailed analyses concerning the key bacterial community were restricted to the rhizosphere subset data. Relative abundance for each ASV was derived by dividing its count by the total count per sample. To visualize the overall community composition, square bar plots were generated, depicting highly abundant taxa across phylum, genus, and ASV levels based on their cumulative relative abundance across the entire dataset. Differentially abundant taxa influenced by disease condition and soil origin were evaluated by ANCOM-BC2 (Analysis of Compositions of Microbiomes with Bias Correction) (v2.10.1) (Lin and Das Peddada, 2020) with *p*-value correction applied via the Benjamini-Hochberg method. This analysis was performed on the rhizosphere data set, which was pre-filtered to exclude rows that contained zero ASVs across all samples. ANCOM-BC2 calculated the bias-corrected log-fold change (LFC) for each taxon, which serves as a metric for the ratio of abundance between conditions (i.e., soil A vs. soil B, or healthy vs. diseased status) on a logarithmic scale. The output identifies structural zeros (sz): ASVs that are completely absent in one condition but exclusively present in the other.

#### 2.10.2 Analysis of *in planta* experimental data

Results from the initial screening were used for down-selection of antagonists and are interpreted as exploratory, whereas statistical inference and main conclusions are based on the confirmatory trial. All *in planta* data were analyzed in R (version 4.5.1) using generalized linear mixed models (glmmTMB). Disease severity (percentage diseased leaf area) was averaged per plant and modeled using a Gamma error distribution with log link. This model was selected because severity data was continuous, non-negative, and right-skewed, and because the log link ensured strictly positive fitted values and generated multiplicative treatment effects. A small constant was added to accommodate zero values. Treatment was included as a fixed effect, and experimental repetition was included as a random intercept to account for run-to-run variability. The infected control (Diseased control) was set as the reference level to estimate treatment effects relative to the disease baseline. Treatment effects are reported as ratios (treated/control) and percent disease reduction with 95% confidence intervals (Supplementary Excel File 3). Model adequacy was assessed using DHARMa simulation-based residual diagnostics (uniformity, dispersion, outliers). The QQ plot indicated no significant deviation from the expected residual distribution (Supplementary Figure 6). Contrasts versus the diseased control were computed from model-based estimated marginal means and *p*-values were adjusted for multiple testing using Benjamini–Hochberg (α = 0.05). The complete raw data and corresponding R scripts, which contain all necessary parameters and data to regenerate figures, are openly available in a designated GitHub repository (see text footnote 4)).^5^

## 3 Results

### 3.1 Soil origin and tomato response to late blight

As described in detail in our previous work (Orwa et al., 2025), the two soils were characterized as loam soils with comparable physicochemical characteristics. The two soil origins responded differently to inoculation with *P. infestans*. In particular, plants in both soils exhibited disease symptoms, but those in soil A had significantly lower fresh weight compared to the healthy control (Supplementary Figure 2).

### 3.2 Identification of broad-spectrum antagonistic bacteria in disease-stable tomato rhizosphere

A collection of 594 bacterial isolates was established with a proportionate distribution across the two soil origins: soil A contributed 302 isolates (50.8%), and Soil B contributed 292 isolates (49.2%). The initial screening regarding *in vitro* antagonistic activity against *P. infestans* and *A. solani* resulted in 116 bacterial isolates (19.2% of the total collection) with antagonistic activity against *P. infestans* and 93 isolates (15.7%) demonstrating antagonism against *A. solani* (Supplementary Excel File 1). Soil B isolates exhibited approximately twice the rate of activity against *P. infestans* (25.0%, *n* = 73) and nearly three times the activity against *A. solani* (24.3%, *n* = 71) compared to soil A (13.6%, *n* = 41 and 7.3%, *n* = 22, respectively).

**FIGURE 2:**
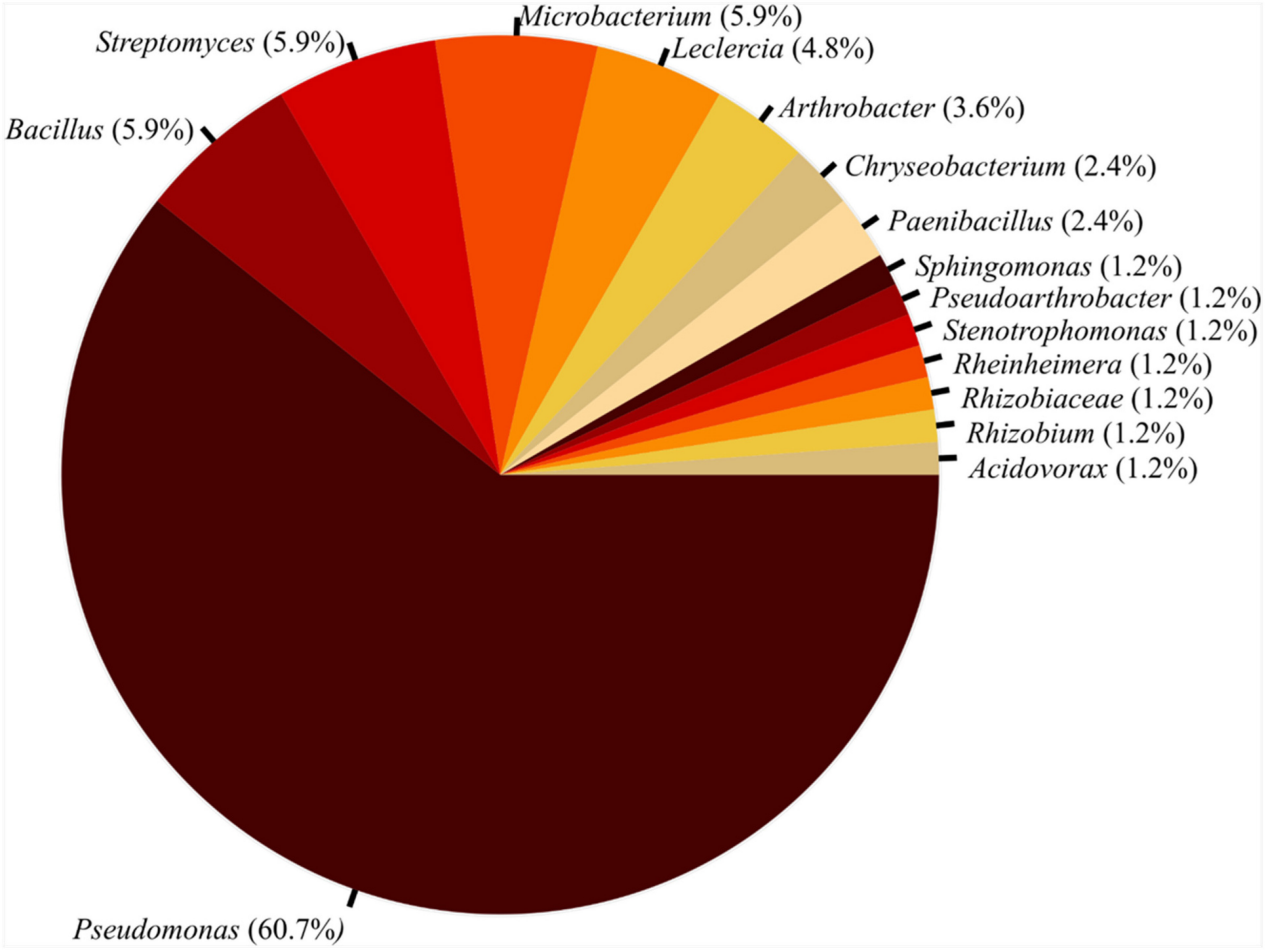
Taxonomic distribution of bacterial isolates in the culture collection inhibiting *Phytophthora infestans in vitro*. The genus determination was obtained through BLAST analysis after partial sequencing of the 16S rRNA gene locus, *N* = 84.

From the primary screening, 116 bacterial strains that exhibited *in vitro* inhibition of *P. infestans* and/or *A. solani*, 84 antagonistic candidates were retained after eliminating strains that lost viability or inhibitory effects during repeated screenings. Taxonomic identification of these 84 isolates based on 16S rRNA sequencing identified *Pseudomonas* as the dominant genus (approximately 60.7% of total isolates), followed by *Bacillus, Streptomyces*, and *Microbacterium* with 5.9% each genus (Figure 2). The identified candidate antagonists were then analyzed for broad-spectrum activity by expanding the screening toward activity against the bacterial pathogens *P. syringae* pv. *tomato* and *X. vesicatoria*. Of the 84 isolates inhibiting *P. infestans*, 20 strains inhibited *P. syringae* pv. *tomato* and 51 strains inhibited *X. vesicatoria*, revealing broad-spectrum activity (Supplementary Excel File 2). These candidates included several strains from the genera *Pseudomonas* and *Bacillus* as well as selected representatives of the genera *Paenibacillus*, *Chryseobacterium*, and *Streptomyces*.

The final screening step, in which we excluded several higher-represented genera (including *Pseudomonas, Bacillus*, and *Streptomyces*) and genera known to contain plant-, human-, or animal-pathogenic species, included 28 strains representing the full taxonomic and ecological diversity of the initial antagonist collection. These 28 isolates were subjected to an expanded *in vitro* inhibition assay against three additional fungal pathogens (*Rhizoctonia solani*, *Fusarium oxysporum, Botrytis cinerea*). *Pseudomonas* consistently emerged as the most versatile genus displaying strong suppression of multiple fungal pathogens and, in some cases, both bacterial pathogens (Table 1). Additional high-performing strains included *Bacillus*, *Paenibacillus*, *Pseudarthrobacter*, and *Streptomyces*, which exhibited broad-spectrum antifungal activity.

**TABLE 1:** *In vitro* inhibitory effects of bacterial antagonists against bacterial and fungal pathogens of tomato.

Bacterial isolates	*P. infestans*	*A. solani*	*R. solani*	*F. oxysporum*	*B. cinerea*	*P. syringae*	*X. vesicatoria*
*Acidovorax* sp._Pb575	++	−	+	−	+	−	−
*Bacillus* sp._Pb109	++	−	−	−	−	−	+
*Bacillus* sp._Pb168	+++	+	+	+	++	−	++
*Bacillus* sp._Pb396	++	+	+	−	++	−	−
*Bacillus*sp._Pb444	+++	+	+	−	++	+	+
*Chryseobacterium* sp._Pb107	++	−	−	−	−	−	+
*Microbacterium* sp._Pb539	+	−	+	+	++	−	+
*Paenibacillus* sp._Pb160	++	+	+	+	++	−	++
*Paenibacillus* sp._Pb355	++	−	+	−	+	−	+
*Pseudarthrobacter* sp._Pb177	++	+	+	+	++	−	++
*Pseudomonas* sp._Pb114	++	+	+	+	+	−	++
*Pseudomonas* sp._Pb151	+++	+	+	−	+	+	++
*Pseudomonas* sp._Pb16	++	+	+	−	++	−	−
*Pseudomonas* sp._Pb170	+++	+	+	+	+	−	++
*Pseudomonas* sp._Pb176	++	+	+	−	+	−	++
*Pseudomonas* sp._Pb271	++	+	+	+	+	++	++
*Pseudomonas* sp._Pb285	++	+	+	+	+	−	−
*Pseudomonas* sp._Pb29	++	+	+	−	−	++	++
*Pseudomonas* sp._Pb377	++	+	+	−	+	−	++
*Pseudomonas* sp._Pb457	++	+	+	+	++	−	++
*Pseudomonas* sp._Pb460	++	+	−	−	−	−	++
*Pseudomonas* sp._Pb521	++	+	+	+	+	nt	++
*Rhizobiaceae* sp._Pb565	+	−	+	−	−	+	+++
*Rhizobium* sp._Pb242	++	+	+	+	++	+	−
*Sphingomonas* sp._Pb171	+	+	+	+	+	−	++
*Streptomyces* sp._Pb301	++	+	+	+	+	−	+
*Streptomyces* sp._Pb39	++	+	+	+	++	−	−
*Streptomyces* sp._Pb557	+	−	+	+	+	−	+

The scores for inhibitory activity represent the average measure of halo diameter from three replicate assays. Key: > above 4 cm (+++): very strong inhibition; 2–3 cm (++): strong inhibition; < 2.0 cm (+): moderate/weak inhibition; 0 cm (-): no inhibition; nt: not tested. The tests were performed in three technical replicates. In the table, we show bacterial antagonists tested against the following tomato pathogens: *Phytophthora infestans*; *Aternaria solani*; *Rhizoctonia solani*; *Fusarium oxysporum*; *Botrytis cinerea, Pseudomonas syringae* pv. *tomato* and *Xanthomonas vesicatoria.*

### 3.3 Siderophore and lytic enzyme activity of the candidate bacterial biocontrol isolates

**FIGURE 3:**
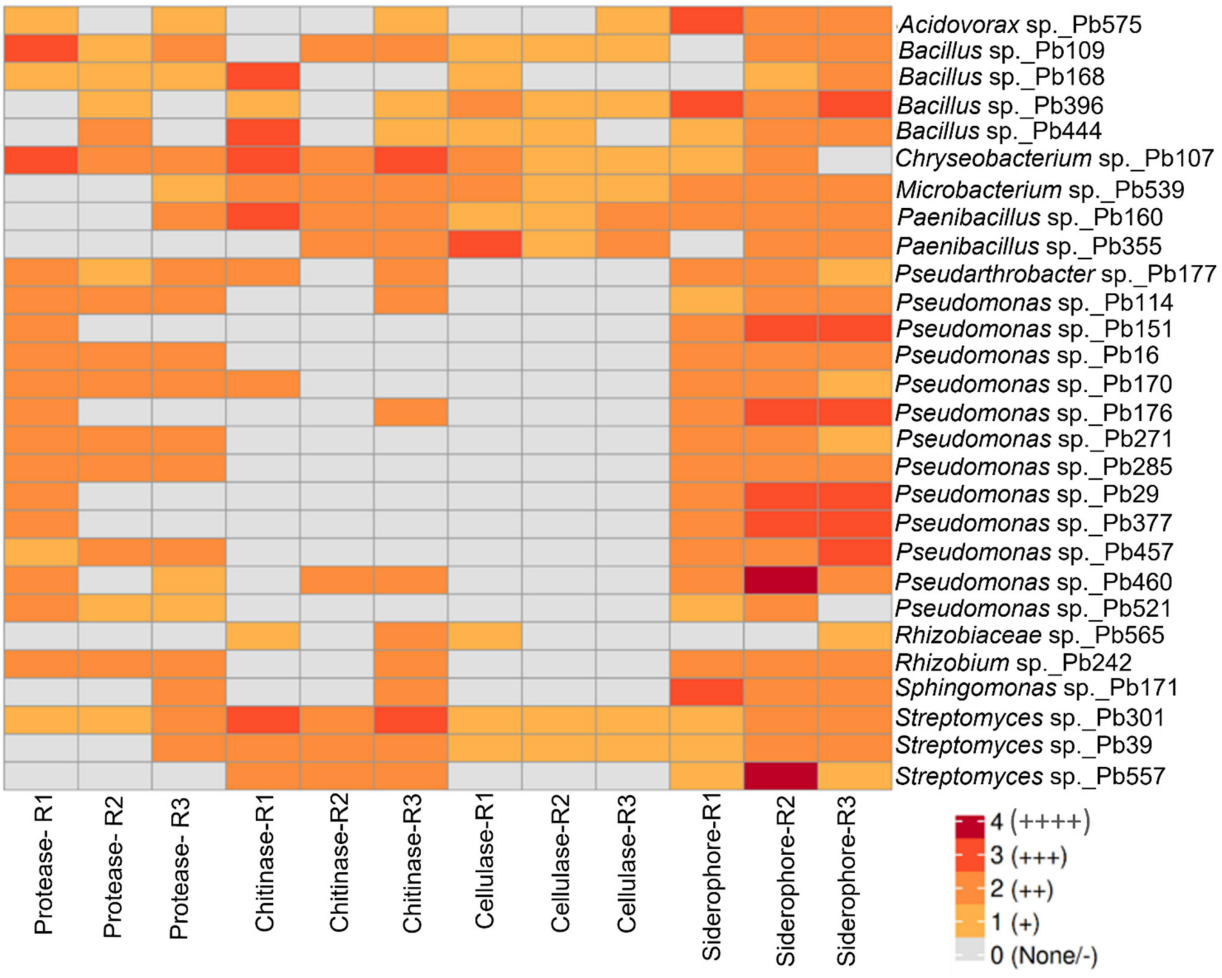
A heatmap showing activity of extracellular lytic enzymes (protease, chitinase, cellulase) and siderophores by selected antagonistic bacteria. Results of three replicate assays with three spots each replicate are shown (R1-R3). The level of activity is shown by different color intensities from a scale of 0–4 based on the size of halos, 0 (-): no activity, 1 cm (+): low activity; 2 cm (++): moderate activity; 3 cm (+++): high activity; 4 cm (++++): very high activity. For the activity, the average value of the size of the halo zone was determined from the three spots per Petri dish.

Functional screening of the 28 selected bacterial antagonists revealed enzymatic and siderophore activity of several genera (Figure 3). Siderophore activity was detected in nearly all strains, with 13 isolates expressing moderate to strong activity. A majority of strains from the genera *Streptomyces, Paenibacillus, Microbacterium, Chryseobacterium*, and *Bacillus* demonstrated moderate to strong and broad activity for proteases, chitinases, and cellulases. The genera *Acidovorax, Planomicrobium, Pseudarthrobacter*, and *Sphingomonas* tended to exhibit a more specialized profile, often showing high activity in one or two classes of lytic enzymes tested. Lower enzyme activity was evident in the *Pseudomonas*, *Rhizobium*, and *Rhizobiaceae* strains, which were characterized by a profile consistently lacking cellulase activity and exhibiting weak or inconsistent chitinase production.

### 3.4 Disease suppressive efficacy of selected bacterial isolates against *P. infestans* in tomato *in planta*

In the initial exploratory *in planta* screening, a clear difference in disease level between non-treated (diseased control) and copper-treated plants was observed, with copper-treated plants having only 15% disease symptoms vs. non-treated plants (diseased control) showing 60% disease severity (Supplementary Figure 3). This finding confirmed the reliability of the established pathogenicity bioassay. Most of the 28 candidate isolates showed the tendency to suppress *P. infestans* compared to the diseased control. Particular strains from *Pseudomonas, Streptomyces*, and *Pseudarthrobacter* reduced disease severity to below 10–15%, in some cases even exceeding the disease-level reduction of the copper treatment. This preliminary assay was conducted with three plants per treatment using a single bacterial suspension and was therefore intended for qualitative comparison and candidate prioritization rather than statistical inference.

**FIGURE 4:**
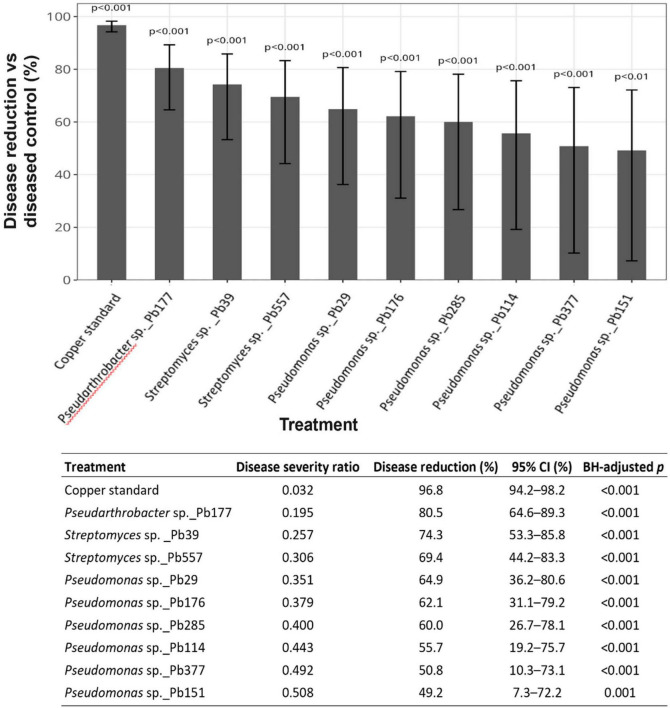
Model-based estimates of *Phytophthora infestans* disease severity reduction by nine bacterial isolates in tomato plants. Bacterial strains were applied preventively to leaves of intact tomato plants as standardized suspensions adjusted to 0.2 OD650, 24 h prior to inoculation with *P. infestans* (2 × 10^4^ sporangia mL ^–1^). Plants treated with distilled water served as the non-inoculated negative control (Healthy control), while plants infected with *P. infestans* alone served as the positive control (Diseased control). A chemical control was included by applying Cuprozin progress (0.52%) prior to pathogen inoculation (Copper standard). Each treatment consisted of six tomato plants (*n* = 1). Disease severity, expressed as the percentage of diseased leaf area, was assessed 5 days post inoculation. Dots on the forest plots represent estimated percentage reduction in disease severity for each treatment, derived from a generalized linear mixed model (GLMM), and vertical lines indicate 95% confidence intervals (CI). Estimates are shown relative to the diseased control and significance was assessed using Benjamini–Hochberg–adjusted (*p* ≤ 0.05). Results are shown for three replicate experiments.

Nine bacterial antagonists with the most promising disease suppressive effects were subjected to the second round of plant trials with six tomato plants in three replicates (Figure 4). Model-based estimates from the generalized linear mixed model revealed that all tested bacterial treatments significantly reduced tomato late blight severity compared to the diseased control (BH-adjusted *p* < 0.01). Disease severity under copper treatment was reduced by > 95%, confirming assay validity. Among bacterial antagonists, *Pseudarthrobacter* sp._Pb177 showed the strongest suppression, reducing disease severity by 80.5%. *Streptomyces* isolates Pb39 and Pb557 also conferred substantial protection (74.3 and 69.4% reduction, respectively). *Pseudomonas* isolates displayed moderate but more variable effects, with mean disease reductions ranging from 49.2 to 64.9%, as reflected by wider confidence interval (CIs).

### 3.5 Bacterial rhizosphere community varies in composition between healthy and late-blight infected tomato plants

Bacterial alpha diversity metrics, including richness, Shannon diversity index, evenness, and the Simpson index, were higher in the rhizosphere and the phyllosphere of soil A than in soil B, reflecting higher bacterial diversity associated with soil A (Supplementary Figure 4). Significant differences in bacterial Shannon diversity, Simpson index, and evenness between healthy and *P. infestans-*infected samples were detected exclusively in the rhizosphere of soil B, with no corresponding effects observed in the endosphere and phyllosphere (Supplementary Figure 4). Shannon diversity in the phyllosphere was higher in soil A than in soil B, with disease status further differentiating healthy and *P. infestans*-infected samples (Supplementary Figure 4).

**FIGURE 5:**
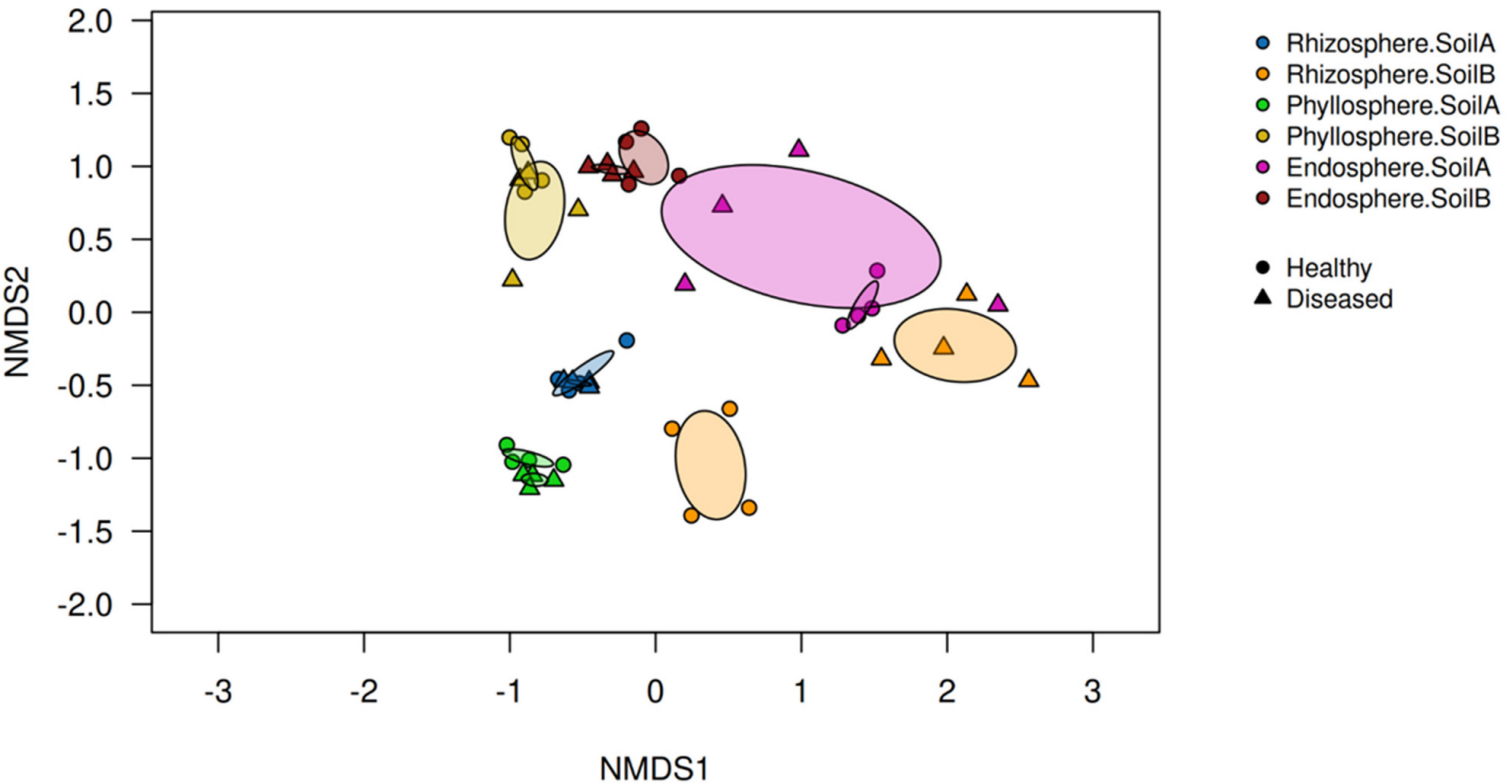
Bacterial beta diversity across tomato microcompartments visualized by non-metric multidimensional scaling (NMDS) based on Bray-Curtis community dissimilarities. Samples were collected from the rhizosphere, phyllosphere, and endosphere of healthy and *Phytophthora infestans-*infected tomato plants (cultivar ‘Red Robin’) grown in two soil origins (soil A and soil B) 14 days post inoculation. Significant differences in community composition were assessed using ANOSIM (*p* ≤ 0.001).

Beta diversity analysis based on Bray-Curtis dissimilarities revealed that bacterial community composition was primarily structured by plant microcompartment, with clear separation among rhizosphere, phyllosphere, and endosphere samples (Figure 5). Soil origin further contributed to differences in beta diversity, particularly in the rhizosphere and phyllosphere, where communities from soils A and B showed stronger separation than in the endosphere. In contrast, *P. infestans* infection did not cause a clear overall clustering of bacterial communities, although a separation between healthy and diseased samples was evident in the rhizosphere of soil B. These results indicate that disease effects on beta diversity were weaker and more context-dependent than those of soil origin and microcompartment.

### 3.6 Soil origin drives rhizosphere bacterial community composition

Across the entire dataset, 11,497 amplicon sequence variants (ASVs) were detected. Of these, 7,429, 2,293, and 6,344 ASVs were observed in the phyllosphere, endosphere, and rhizosphere, respectively, with substantial overlap of ASVs among the compartments. Based on structural zero analysis, 2,272 ASVs were shared between healthy and diseased rhizosphere samples, while 2,444 ASVs were unique to healthy plants and 1,628 ASVs were unique to diseased plants.

**FIGURE 6:**
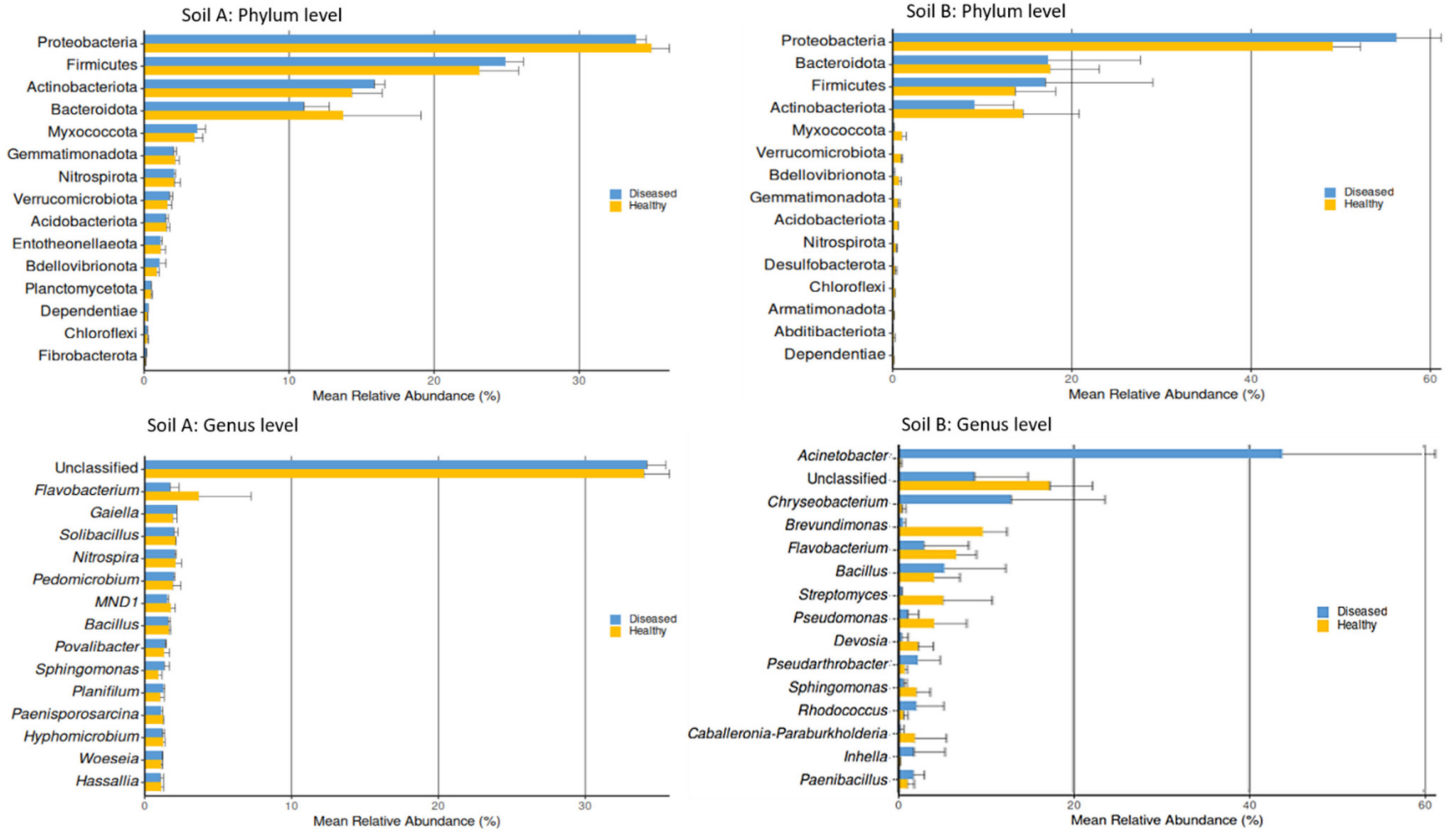
Top-abundant phyla and genera between the rhizosphere of soil A and soil B under healthy and *Phytophtora infestans*-infected conditions of tomato plants (cultivar ‘Red Robin’). Bars represent the mean relative abundance (%) of each taxon. Error bars indicate standard deviation. No significant difference was observed in taxa abundance between samples from soil origins A and soil B, inferred at *p*- ≤ 0.05 according to Benjamini-Hochberg correction, which controls for false discovery rate, analyzed using ANCOM-BC2 (Analysis of Compositions of Microbiomes with Bias Correction).

Because the rhizosphere showed the strongest microbial response to *P. infestans* infection (Figure 6 and Supplementary Figure 4), subsequent abundance analyses focused on this compartment to resolve pathogen-associated taxonomic and abundance shifts in both soil origins. Relative abundance profiling of the rhizosphere taxa revealed strong soil-dependent differences in bacterial community composition at both the phylum and genus levels (Figure 6). In both soils, Proteobacteria dominated the rhizosphere, followed by Firmicutes, Actinobacteriota, and Bacteroidota; however, their relative contributions differed markedly between soil A and soil B. At the genus level, soil A was characterized by higher relative abundances of *Flavobacterium*, *Gaiella*, and several unclassified taxa, whereas soil B showed a pronounced enrichment of *Acinetobacter*, *Chryseobacterium*, and *Brevundimonas*. While disease condition led to moderate shifts in the relative abundance of specific taxa within each soil, the overall bacterial community composition remained primarily structured by soil origin.

**FIGURE 7:**
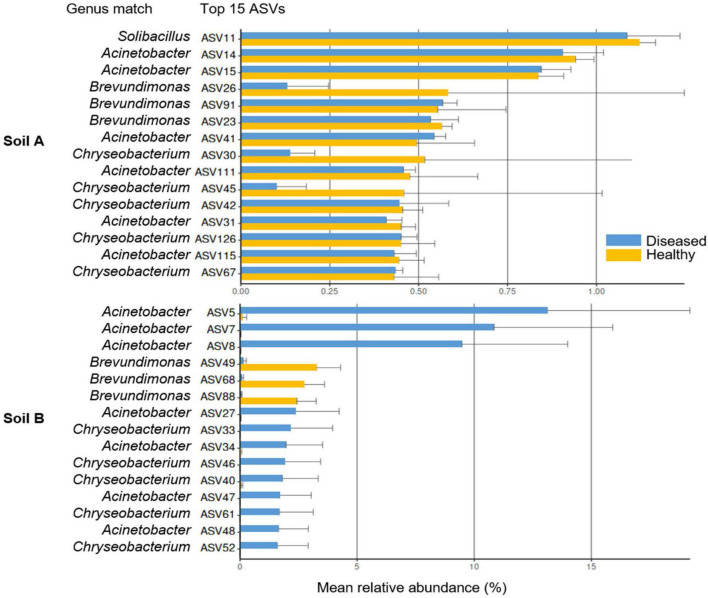
Top-abundant taxa between the rhizosphere of soil A and soil B at the ASV level under healthy and *Phytophthora infestans*-infected conditions of tomato plants (cultivar ‘Red Robin’). Bars represent the mean relative abundance (%) of each taxon. Error bars indicate standard deviation. No significant difference in taxa abundance between samples from soil origins A and soil B, inferred at *p*- ≤ 0.05 according to Benjamini-Hochberg correction, which controls for false discovery rate, analyzed using ANCOM-BC2 (Analysis of Compositions of Microbiomes with Bias Correction).

At the ASV level, bacterial communities in the rhizosphere were structured based on soil origin and disease status of the plants, reflecting the dominance of distinct taxa (Figure 7). In soil A, the top-ranked ASVs were assigned to the genera *Solibacillus, Acinetobacter*, *Brevundimonas*, and *Chryseobacterium.* ASV11 (*Solibacillus*) and ASV14–15 (*Acinetobacter*) accounted for a substantial proportion of the total relative abundance in both healthy and diseased samples. In soil B, the top-ranked ASVs were linked to the genera *Acinetobacter*, *Brevundimonas*, and *Chryseobacterium.* ASV5, ASV7, and ASV8, all assigned to *Acinetobacter*, accounted for a substantial proportion of the total relative abundance, but primarily in diseased samples. Notably, ASV 49, ASV68, and ASV 88, all belonging to the genus *Brevundimonas*, were the three most abundant ASVs in healthy samples of soil B (Figure 7).

Although relative-abundance bar plots suggested differences among top-ranked rhizosphere taxa (soil B) with reference to plant health status, these patterns were not supported by statistical testing. Differential-abundance analysis based on ANCOM-BC2 revealed that the patterns reflected within-group variability rather than consistent shifts between disease states, resulting in no statistically significant differences among dominant taxa.

### 3.7 Cultured antagonists correspond to top-ranked and stable rhizosphere taxa

A substantial proportion of the genera represented of the viable antagonistic isolates also belonged to the top-ranked taxa in the 16S rRNA microbiome dataset, including *Chryseobacterium, Bacillus*, *Paenibacillus, Pseudomonas*, and *Streptomyces*, across microcompartments, plant health states, and soil origin (Figure 6 and Supplementary Figure 5).

**FIGURE 8:**
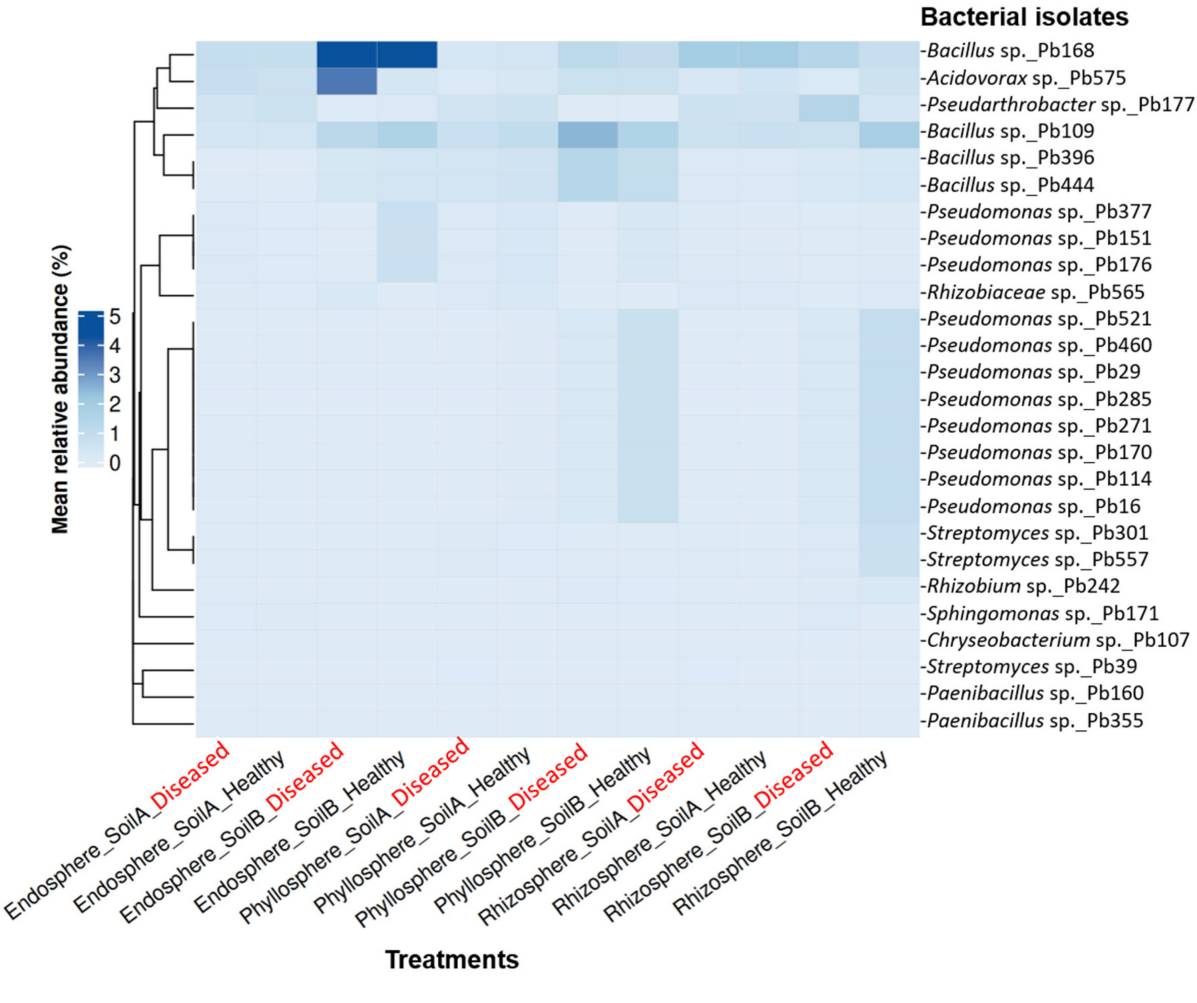
Mapping of antagonistic bacterial isolates to detected ASVs in the tomato microbiome. Heatmap showing the mean relative abundance (%) of amplicon sequence variants (ASVs) corresponding to antagonistic bacterial isolates across tomato (cultivar ‘Red Robin’) endosphere, phyllosphere, and rhizosphere samples from soil A and soil B under healthy and *Phytophthora infestans*–infected conditions. Relative abundances were calculated from normalized 16S rRNA amplicon profiles and averaged within each compartment, soil, and health status. Rows represent bacterial isolates and columns represent treatments (experimental groups). Color intensity reflects mean relative abundance (0–5%). Isolates were hierarchically clustered using Bray-Curtis dissimilarity to highlight similarities in abundance profiles across compartments. Isolate 16S rRNA gene sequences (∼900 bp) were matched to ASVs (∼250 bp) using BLASTn with 100% sequence identity and ≥ 50% query coverage.

To link the viable bacterial isolates with the total microbial community, the 16S rRNA-sequences from our final collection of 28 proven antagonists were mapped to their corresponding ASVs detected in the microbiome dataset (Figure 8). Although top-ranked genera account for a substantial fraction of the community, individual isolate-associated ASVs occurred at low relative abundance across samples, resulting in low mean values for the 28 antagonistic isolates (Figure 8). Notably, these ASVs were repeatedly detected across compartments and soils, indicating that antagonistic isolates correspond to recurrently detected microbiome members rather than sporadically observed taxa. Hierarchical clustering of the isolate-associated ASV profiles showed that their abundance patterns were structured primarily by microcompartment and soil origin, with no consistent separation by disease status (Figure 8). Furthermore, we detected more *Pseudomonas*-associated ASVs in healthy than in diseased samples. *Pseudarthrobacter*-associated ASVs, corresponding to the most consistently effective antagonist *in planta*, showed no strong compartment or soil specificity. In contrast, *Bacillus-* and *Pseudomonas*-associated ASVs displayed clear enrichment patterns in soil B, with *Bacillus* enriched in the endosphere and phyllosphere and *Pseudomonas* enriched in the phyllosphere and rhizosphere (Figure 8).

## 4 Discussion

Sustainable management of plant pathogens, including *P. infestans*, depends increasingly on understanding the role the resident microbial communities play in protecting the host against disease (Vannier et al., 2019). While next-generation sequencing has revolutionized our ability to characterize plant-associated microbiomes, culture-dependent approaches remain indispensable for isolating microbial antagonists (Anzalone et al., 2021; Lee et al., 2016; Quiza et al., 2015). Here, we combined culture-based isolation, multi-stage antagonism assays, and functional profiling of lytic enzymes and siderophore activity with 16S rRNA gene sequencing analysis of the bacterial community. The aim was to identify ecologically relevant bacterial antagonists (including *Pseudarthrobacter*, *Pseudomonas*, and *Streptomyces* sp.) of tomato late blight isolated from healthy and *P. infestans*-infected tomato plants grown in different soil environments.

Culturable isolates are a prerequisite for evaluating their biocontrol potential against plant pathogens, providing a practical basis for reducing reliance on synthetic pesticides in sustainable agriculture. Consistent with previous studies that abundance and diversity of antagonistic microorganisms vary across soils and plant production sites (Anzalone et al., 2021; Caulier et al., 2018; Marín-Guirao and de Cara-García, 2025), we observed soil-specific differences in the antagonistic potential of bacterial taxa. Specifically, isolates inhibiting *P. infestans*, *A. solani*, and other tomato pathogens were enriched in soil B, suggesting a more selective microbial environment that favors bacteria with competitive or inhibitory traits through ecological filtering (Causevic et al., 2025; Elsayed et al., 2021; Zheng et al., 2021). *Pseudomonas* emerged as the most abundant and versatile genus, showing broad inhibitory activity against multiple fungal and bacterial tomato pathogens, supporting its established role in biocontrol (Dimkiæ et al., 2022; Müller and Behrendt, 2021; Topalović et al., 2020; Zheng et al., 2021). Additional genera, including *Bacillus*, *Paenibacillus, Streptomyces*, *Microbacterium*, and *Pseudarthrobacter*, harbor important antagonists. Members of these genera are widely reported as broad-spectrum biocontrol agents against phytopathogens (Dobrzyński and Naziêbło, 2024; Fira et al., 2018; Lee et al., 2023; Marín-Guirao and de Cara-García, 2025; Solanki et al., 2022; Sui et al., 2022; Sun et al., 2025; Wang et al., 2025).

Functional characterization of our bacterial collection indicates that antagonistic potential is linked to distinct biocontrol-related traits (Admassie et al., 2022; Ali et al., 2020; Saberi Riseh et al., 2024), with taxon-specific patterns of lytic enzyme activities (Admassie et al., 2022; Ajuna et al., 2023; Saberi Riseh et al., 2024; Tariq et al., 2025). For example, *Streptomyces, Paenibacillus, Microbacterium, Chryseobacterium*, and *Bacillus* displayed broader and more intense profiles for proteases, chitinases, and cellulases, while other genera, such as *Pseudomonas*, exhibited more restricted activity. Thus selected antagonists differ in their biocontrol-associated functional traits, and the soil origin influences both the taxa recovered and the biocontrol-related traits expressed among culturable bacteria, confirming previous studies (Nishisaka et al., 2025; Suarez-Fernandez et al., 2025).

Antagonism shown in *in vitro* experiments often does not predict disease control *in planta* or in soil. Rapid *in vitro* assays select mainly for antibiosis, missing key traits such as root or leaf colonization and plant defense induction (Bonaterra et al., 2022). In line with functional screening, the results of our *in planta* assays revealed that all our antagonistic isolates translated *in vitro* activity into consistent disease suppression against *P. infestans*. Bacterial strains, particularly from the genus *Pseudarthrobacter*, significantly reduced disease severity *in planta* by 60–80% relative to diseased control. However, the magnitude of disease suppression varied among the taxa, particularly for *Pseudomonas* and *Streptomyces*, consistent with reported variation in host association and *in planta* antagonistic stability among biocontrol agents (Lv et al., 2023; Sun et al., 2025). A broader review of plant beneficial microbes for late blight management is dominated by bacterial genera, including *Pseudomonas*, *Bacillus*, *Streptomyces*, and fungal genera *Trichoderma*, *Penicillium*, documented (Hashemi et al., 2022; Oubaha et al., 2021; Wang and Long, 2023). In this study, *Pseudarthrobacter_*Pb177 emerged as the most effective antagonist suppressing *P. infestans* by nearly 80% relative to diseased control. To our knowledge, *Pseudarthrobacter* has not been reported as antagonists of *P. infestans*, identifying this genus as a novel candidate for tomato late blight biocontrol. Existing work highlights strong antagonism of *Pseudarthrobacter polychromogenes* AEND14 against pepper bacterial spot (*Xanthomonas euvesicatoria*), reducing disease severity by nearly 85% in greenhouse experiments (Caplik and Küsek, 2025). Other strains of *Pseudarthrobacter* have also been shown to inhibit *Ralstonia solanacearum* (causative agent for tomato bacterial wilt) when applied as a co-culture with *Lysinibacillus sphaericus* HR92 and inulin (Yan et al., 2025). Current evidence indicates that *Pseudarthrobacter* is not yet used as a stand-alone commercial biopesticide. Instead, it is primarily studied in experimental settings as a promising plant growth-promoting and biostimulant organism with indirect benefits for pest and disease suppression (Ham et al., 2022; Naloka et al., 2024). Future applications in managing phytopathogens remain promising but are at an early research stage. These results demonstrate that effective biocontrol depends not only on antagonistic activity but also on stable and reproducible performance *in planta*, highlighting the importance of multi-stage validation when selecting candidates for further development.

In the 16S rRNA microbiome profiling, diversity analyses of the bacterial community composition revealed soil origin as the dominant driver of tomato-associated bacterial community assembly, particularly in the phyllosphere and rhizosphere. Strong and consistent separation by soil origin in beta diversity analyses indicates that edaphic conditions primarily shape microbiome structure (Anzalone et al., 2022; Cheng et al., 2020; Malacrinò and Bennett, 2024; Zhou et al., 2025). This pattern was further supported by alpha diversity metrics, with soil A exhibiting higher bacterial richness, diversity, and evenness than soil B. A similar soil-driven structuring was reported for fungal beta diversity across both compartments in our previous work (Orwa et al., 2025). Notably, fungal alpha diversity was higher in rhizosphere samples from soil B than from soil A, contrasting with bacterial trends and highlighting distinct bacterial and fungal responses to shared soil conditions. Beyond its known role in shaping plant microbiomes, our results show that soil origin is a critical factor for interpreting disease effects and identifying bacteria with biocontrol potential.

Studies have demonstrated that the disease suppression process is shaped not by single biocontrol microbes acting alone, but by rich and dynamic microbial communities whose interactions collectively limit progression of phytopathogens (Anzalone et al., 2021; Quiza et al., 2015; Vannier et al., 2019). Although *P. infestans* did not broadly restructure the rhizosphere microbiome, disease-associated effects were significant in the rhizosphere of soil B. They reflected changes in relative contributions of a few abundant taxa, particularly *Acinetobacter* and *Chryseobacterium*. Similar disease effects on rhizosphere bacterial communities have been reported in tomato and other hosts. In these cases, pathogen invasion altered diversity and assembly processes in some soil or host backgrounds but left communities in others relatively stable, despite comparable pathogen pressure (Handique et al., 2024; Kuang et al., 2023; Zhou et al., 2021, 2025). In contrast to bacteria, we reported in our previous work that disease-associated effects on fungal diversity were most pronounced in the phyllosphere, particularly in plants grown in soil B (Orwa et al., 2025). Related studies also indicate that bacterial and fungal communities differ in their tissue-specific and functional responses to *P. infestans*, with bacteria responding primarily in the rhizosphere and fungi responding more strongly in aboveground tissues (Ketehouli et al., 2024; Kuang et al., 2023; Kudjordjie et al., 2024).

Studies on disease-suppressive and conducive soils show that disease protection is linked to the enrichment or loss of specific Actinobacteria, Firmicutes, *Flavobacteria*, or *Pseudomonas* lineages, while the broader microbial community remains relatively stable (Kwak et al., 2018; Lazcano et al., 2021; Lee et al., 2021). Several lower abundance taxa (including Myxococcota, Acidobacteriota, *Chloroflexi*, *Nitrospira*, etc.) showed soil- and condition-specific enrichment and were more prevalent in the healthy rhizosphere of soil B, suggesting they may indicate a disease-suppressive state (Figure 6). Previous works have shown that tomato rhizosphere communities comprise conserved key taxa dominated by Proteobacteria, Bacteroidetes, and Acidobacteria across cultivars and soils, with *Acinetobacter*, *Arthrobacter*, *Chryseobacterium*, and *Pseudomonas* as some of the most abundant genera (Cheng et al., 2020; Dong et al., 2019). Interestingly, *Acinetobacter*-associated ASVs increased in diseased plants grown in soil B, whereas *Brevundimonas*-associated ASVs were more prominent in healthy samples, indicating disease-linked redistribution within the stable ASV pool (Figure 6). Most abundant rhizosphere taxa were not significantly differentially abundant with respect to disease status or soil origin. This suggests that the observed shifts in beta diversity and relative-abundance potentially reflect high within-group variability and several structural zeros rather than consistent changes in key taxa. These results indicate resilient key rhizosphere taxa dominated by Proteobacteria, Firmicutes, Bacteroidota, and Actinobacteriota, with consistent presence of genera such as *Acinetobacter*, *Brevundimonas*, *Chryseobacterium, Bacillus*, *Pseudarthrobacter*, *Pseudomonas*, and *Streptomyces* across soils and disease states. Besides, disease effects are primarily confined to rare or conditionally present taxa rather than disrupting key bacterial groups relevant for biocontrol.

The observation that many of our bacterial antagonists of *P. infestans* corresponded to ASVs with low mean relative abundance does not imply low functional relevance. Previous studies have demonstrated that effective biocontrol candidates originate from bacterial groups that are consistently present within the tomato-associated microbiome rather than from sporadic or rare taxa (Fagnano et al., 2025; Nicotra et al., 2024). The low mean relative abundance of isolate-associated ASVs, despite their assignment to the top-ranked genera, indicates that effective biocontrol candidates are not defined by high abundance but by their presence within prevalent and well-established bacterial lineages (Tom et al., 2022). Rare members of the microbial biosphere can induce disproportionate effects on ecosystem function and stability, including in host-associated habitats, despite their low numerical abundance in bulk community profiles (Jousset et al., 2017). Extracellular traits (including siderophore activity, antibiosis, and lytic enzymes), together with niche-specific colonization of microhabitats on roots and leaves can mediate functional impact in plant systems (Gu et al., 2020; Kuzyakov and Blagodatskaya, 2015; Tian et al., 2020). As a result, resident microorganisms may reach high local densities and exert strong antagonistic activity even when their average relative abundance is low. Moreover, our microbiome data indicate that soil origin and microcompartment are the primary filters structuring bacterial communities, whereas disease-associated shifts are limited and context-dependent. This finding provides an ecological explanation for why certain antagonists are consistently recovered and show consistent disease suppression in specific soil-compartment conditions (Anzalone et al., 2022; Fagnano et al., 2025; Wang and Sugiyama, 2020). Consistent with soil- and compartment-level filtering, broadly distributed isolate-linked ASVs such as *Pseudarthrobacter* may reflect greater functional flexibility, whereas the compartment-associated patterns of *Bacillus* and *Pseudomonas* point to more specialized ecological roles (Compant et al., 2019; Pérez-Jaramillo et al., 2018). These patterns support the view that effective biocontrol strains are those that combine antagonistic capacity with ecological compatibility, that is, the ability to persist within the target plant habitat and soil context after introduction (Compant et al., 2019; Moussa and Iasur Kruh, 2025; Nicotra et al., 2024).

Limitations to this study include that 16S rRNA microbiome profiling relied on four biological replicates per soil origin and plant condition, which captures robust shifts but may limit detection of subtle effects, particularly for rare taxa. Culture-based isolation used pooled material within each soil and plant condition group which may reduce replicate-level statistical independence for cultivation outputs; therefore, isolation results are interpreted qualitatively. In addition, we acknowledge that low replication in the exploratory *in planta* screening may reduce statistical power and increase uncertainty around effect size estimates.

In conclusion, integrating 16S rRNA gene microbiome profiling with culture-based isolation links community patterns to functionally validated bacterial antagonists of *Phytophthora infestans* and provides a microbiome-guided framework for managing tomato late blight. By combining bacterial cultivation, functional trait screening, community profiling, and ASV-to-isolate mapping, we bridge bacterial community ecology with applied biocontrol research. This approach can serve as a guideline for other plant-pathogen systems to identify suitable candidates for managing phytopathogens. By linking ecological insights to *in vitro* and *in planta* validation, our workflow highlights candidates with potential for practical application. These strains, when further optimized, can provide strategies aimed at reducing reliance on chemical fungicides.

## Data Availability

Raw reads are deposited at the Sequence Read Archive (https://www.ncbi.nlm.nih.gov/sra) under the BioProject accession number PRJNA1273108. The data and custom R scripts used to generate the figures for this study are openly available on GitHub at https://github.com/Philemonorwa/Phytophthora-infestans-challenged-tomato-microbiome-bacteria.git.

## Footnotes

1
https://usegalaxy.eu/

2
https://benjjneb.github.io/dada2/tutorial.html

3
https://www.ncbi.nlm.nih.gov/sra

4
https://github.com/Philemonorwa/Phytophthora-infestans-challenged-tomato-microbiome-bacteria.git
