# Supplementary material for: Linking microbiome structure to functional analysis identifies resilient Pseudarthrobacter, Pseudomonas, and Streptomyces antagonists of Phytophthora infestans in tomato
Source: Front Microbiol. 2026 May 1;17:1810932. doi: 10.3389/fmicb.2026.1810932 (PMC13177180; doi:10.3389/fmicb.2026.1810932)
Supplement: Supplementary file 4 [file Data_Sheet_1.pdf]

Supplementary Material

1 Supplementary Excel Files

All supplementary Excel files are provided as separate files with the following legend.

Supplementary Excel File 1

Initial collection of 594 bacteria isolated from healthy and diseased tomato tissues of tomato plants grown in soil A and soil B. Isolation parameters include media (nutrient agar and oatmeal agar) and temperature (14, 21, and 28 °C) used for the isolation of bacteria from plant samples. Biocontrol candidates inhibiting *Phytophthora infestans* and *Alternaria solani* based on *in vitro* tests on nutrient agar are shown. 'yes' indicates the presence of an inhibition zone.'no' indicates the absence of an inhibition zone.

Supplementary Excel File 2

Collection of 84 promising antagonistic bacterial candidates inhibiting *Phytophthora infestans* based on initial *in vitro* tests. This collection was retested for *in vitro* inhibition of *P. infestans* using 10% rye agar, plus additional tomato pathogens, *Pseudomonas syringae* pv. tomato, *Xanthomonas vesicatoria*, using nutrient agar. The scores for inhibitory activity represent the average measure of halo diameter from three replicate assays. Key: >above 4 cm (+++): very strong inhibition; 2-3 cm (++): strong inhibition; < 2.0 cm (+): moderate/weak inhibition; 0 cm (-): no inhibition; nt: not tested.

Supplementary Excel File 3

Model-based treatment efficacy of bacterial antagonists on reducing the severity of *Phytophthora infestans in planta*. Effects are reported as treated/control ratios (Gamma-log GLMM; back-transformed) and converted to percent disease reduction relative to the diseased control: % reduction = (1 − ratio) × 100. Confidence intervals are 95%.

2 Supplementary Figures and Tables

All supplementary Figures and Tables are provided below.

2.1 Supplementary Figures

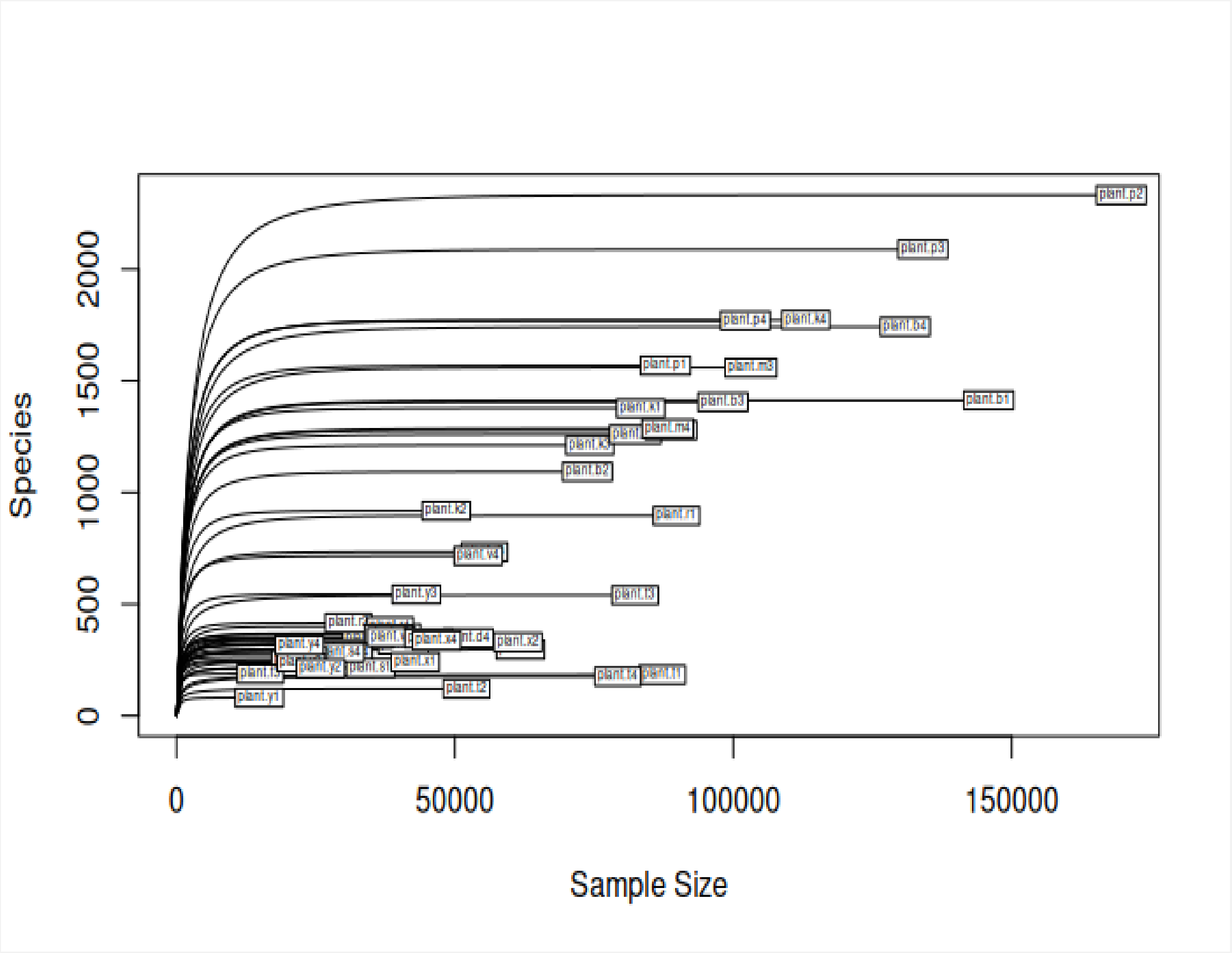

Supplementary Figure 1. Rarefaction curve of observed species richness after sequencing of 16S rRNA gene from the rhizosphere, endosphere, and phyllosphere samples of tomato plants grown in soil origins A and B. The curve was generated by subsampling the dataset multiple times (1000 random rarefaction runs) to estimate ASV distribution at different sequencing depths. All curves reaching saturation indicate sufficient sequencing depth to cover microbial diversity.

.

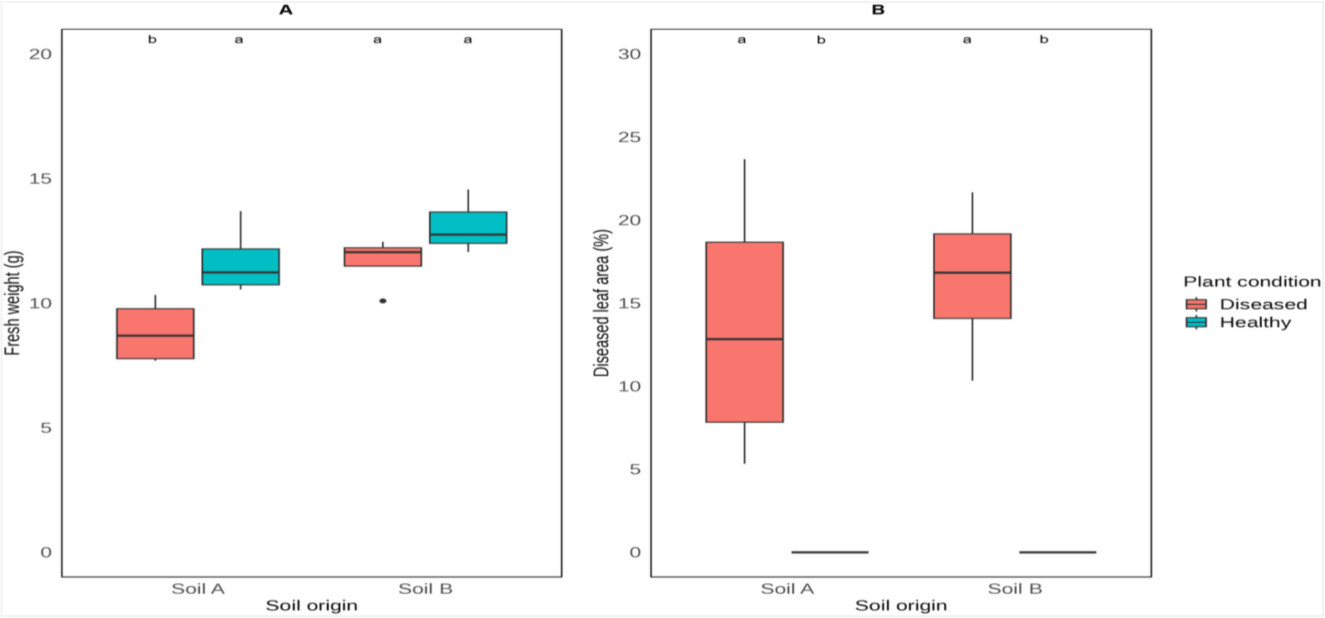

Supplementary Figure 2. Effect of *P. infestans* on tomato plants grown in two distinct organic soil origins (A and B), in terms of (A) plant fresh weight and (B) diseased leaf area (%). Tomato plants were grown in two soils sourced from Domäne Mechthildshausen, Wiesbaden (soil A) and Solidarische Landwirtschaft, Rüsselsheim (soil B). Five weeks post-sowing, plants were challenged with a *P. infestans* conidial suspension (2×10^4 conidia/ml). Tomato plants inoculated with distilled water served as the healthy control. Four tomato plants were used for each treatment (n=4). Data were collected 14 days after inoculation with *P. infestans*. Means were compared with two-way ANOVA ($p \leq 0.01$) followed by Tukey's post-hoc test ($p \leq 0.05$). Significant differences are shown by letters (a, b). This figure is taken from Orwa et al. (2025).

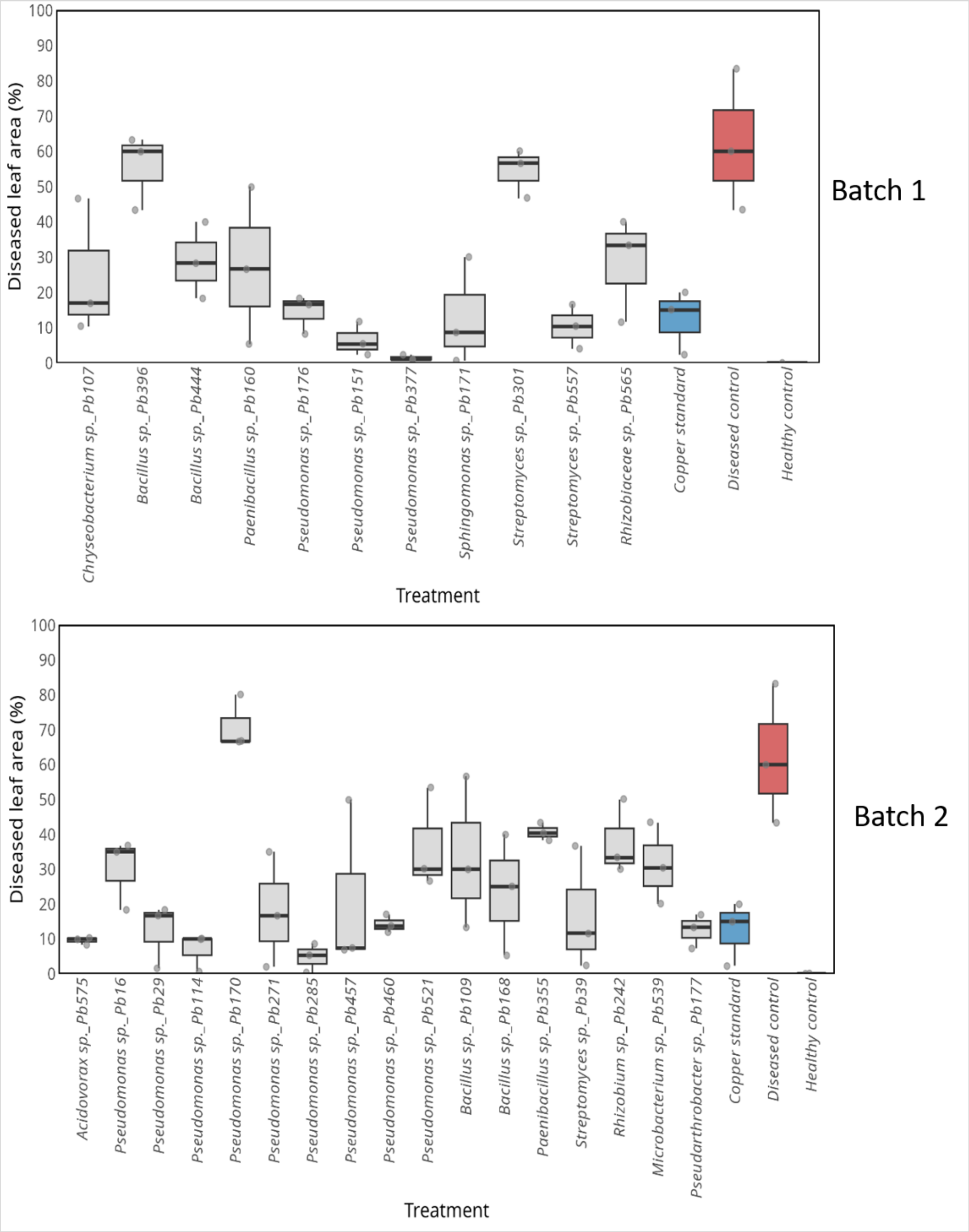

Supplementary Figure 3. Efficacy of 28 selected bacterial isolates in suppressing *Phytophthora infestans* in a one-time screening in the tomato plant cultivar `Red Robin´. Bacterial strains were applied preventively to tomato leaves as standardized suspensions adjusted to OD_{650} 0.2, 24 h prior to inoculation with *P. infestans* (2×10^4 sporangia mL^{-1}). Plants treated with distilled water served as the non-inoculated negative control (Healthy control), while plants infected with *P. infestans* alone served as the positive disease control (Diseased control). As a chemical control, Cuprozin progress (0.52%) was applied (Copper standard), also 24h prior to pathogen inoculation. Each treatment consisted of three tomato plants (n = 3). Disease severity, expressed as the percentage of diseased leaf area, was assessed five days post-inoculation. Data are presented as medians with interquartile ranges (IQR), based on three biological replicates per treatment. Results are shown for one screening trial, divided into two batches due to space limitations.

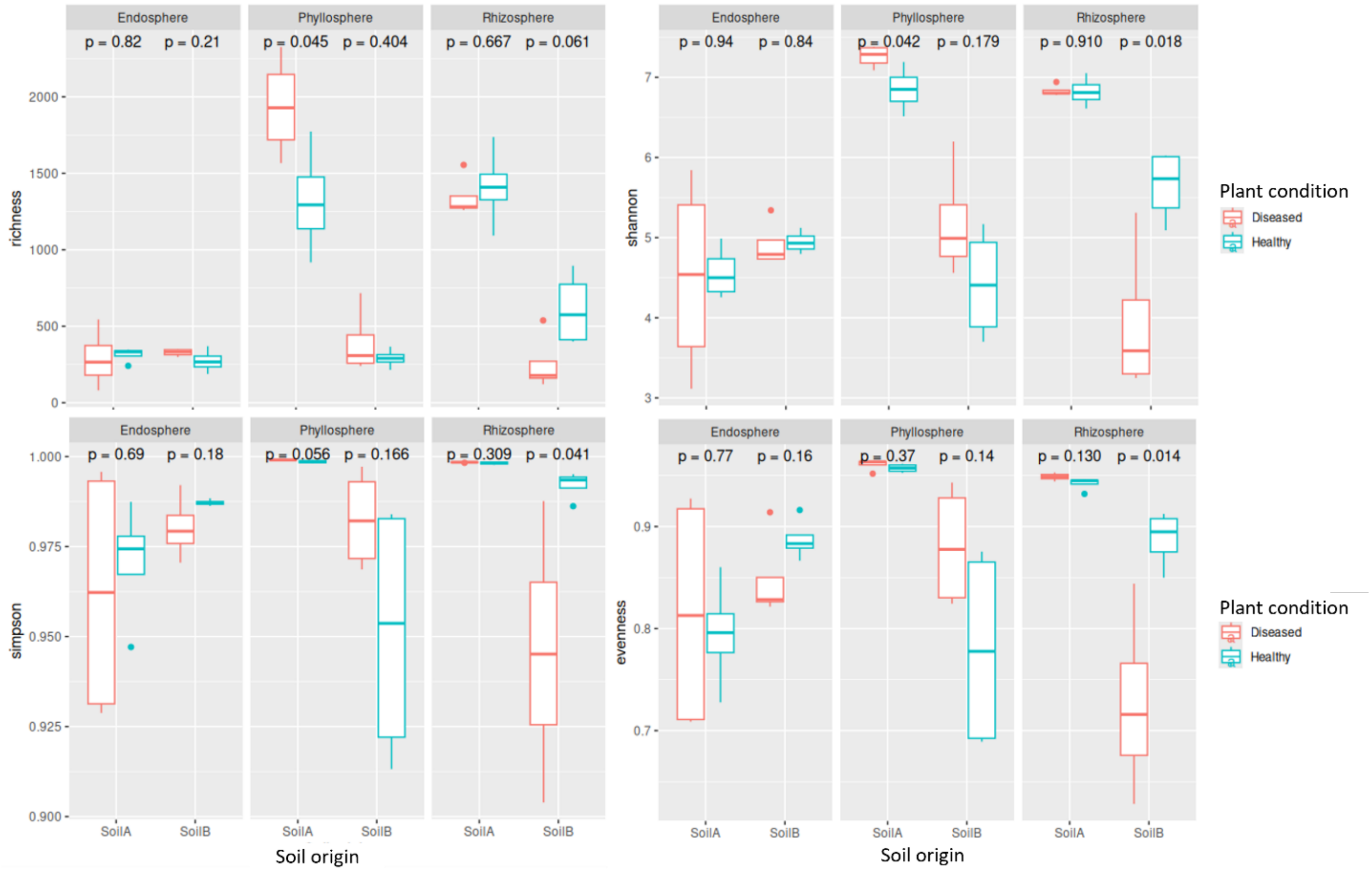

Supplementary Figure 4. The alpha-diversity indices, species richness, Shannon, Simpson, and evenness indices of the bacterial microbiome in samples from the phyllosphere, endosphere, and rhizosphere from tomato plants grown in two soil origins (soil A and soil B). Plants were grown in two different soil origins (A and B) and were inoculated with *P. infestans* (diseased, turquoise plots) or remained untreated (healthy, red plots) 14 days before sampling was done. Four tomato plants were used for each treatment (n=4). Values of alpha-diversity indices are shown, and corresponding p-values are written above each comparison. Means were compared with the Kruskal-Wallis test, with a significance threshold set at $p \leq 0.05$.

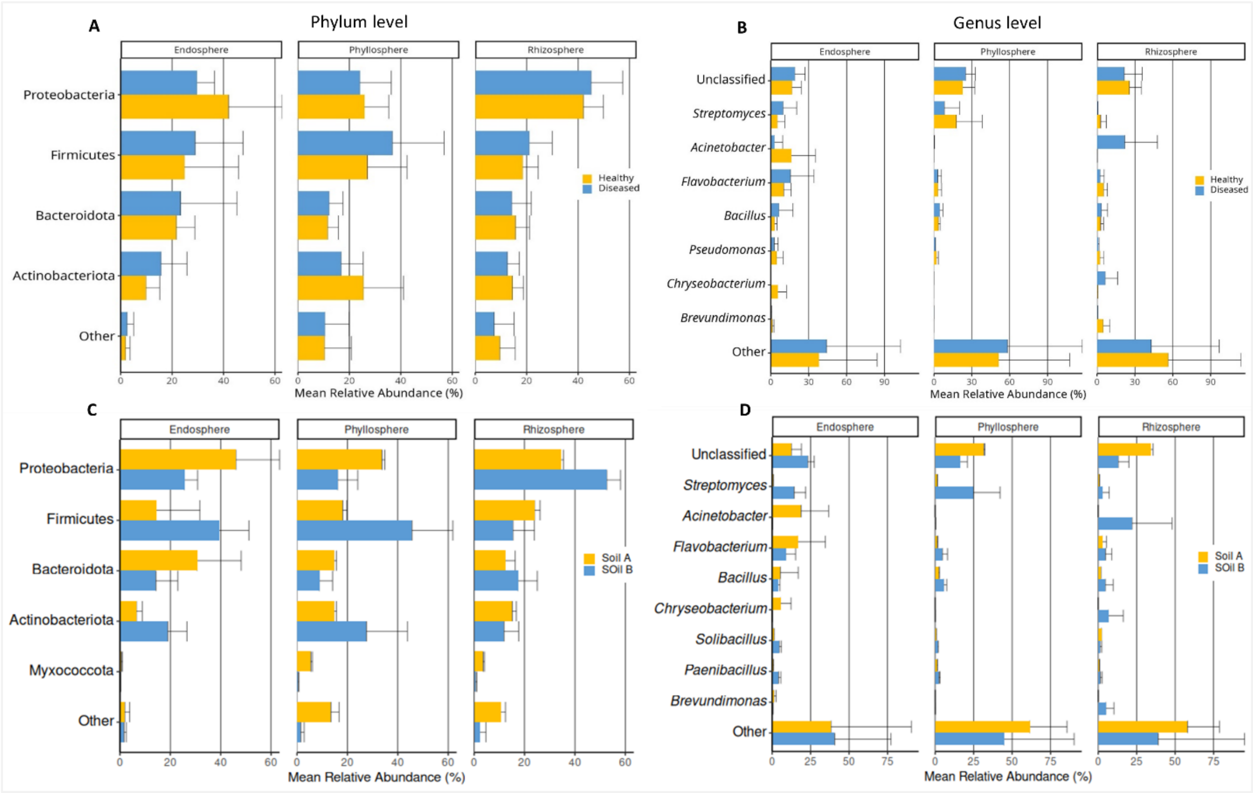

Supplementary Figure 5. Top-ranked bacterial taxa in the whole dataset, considering soil origin and disease status effects. (A, B) Top-ranked bacterial phyla and genera between healthy and *P. infestans* across the endosphere, phyllosphere, and rhizosphere samples. (C, D) Top-ranked bacterial phyla and genera between soil origin A and soil B across the endosphere, phyllosphere, and rhizosphere samples. Bars represent the mean relative abundance (%) of each phylum, genus, and ASV. Others represent all taxa with less than 5% and 3% phylum and genus, respectively, for plant condition, and less than 5% and 4% abundance at phylum and genus levels, respectively, for soil origin. Error bars indicate standard deviation.

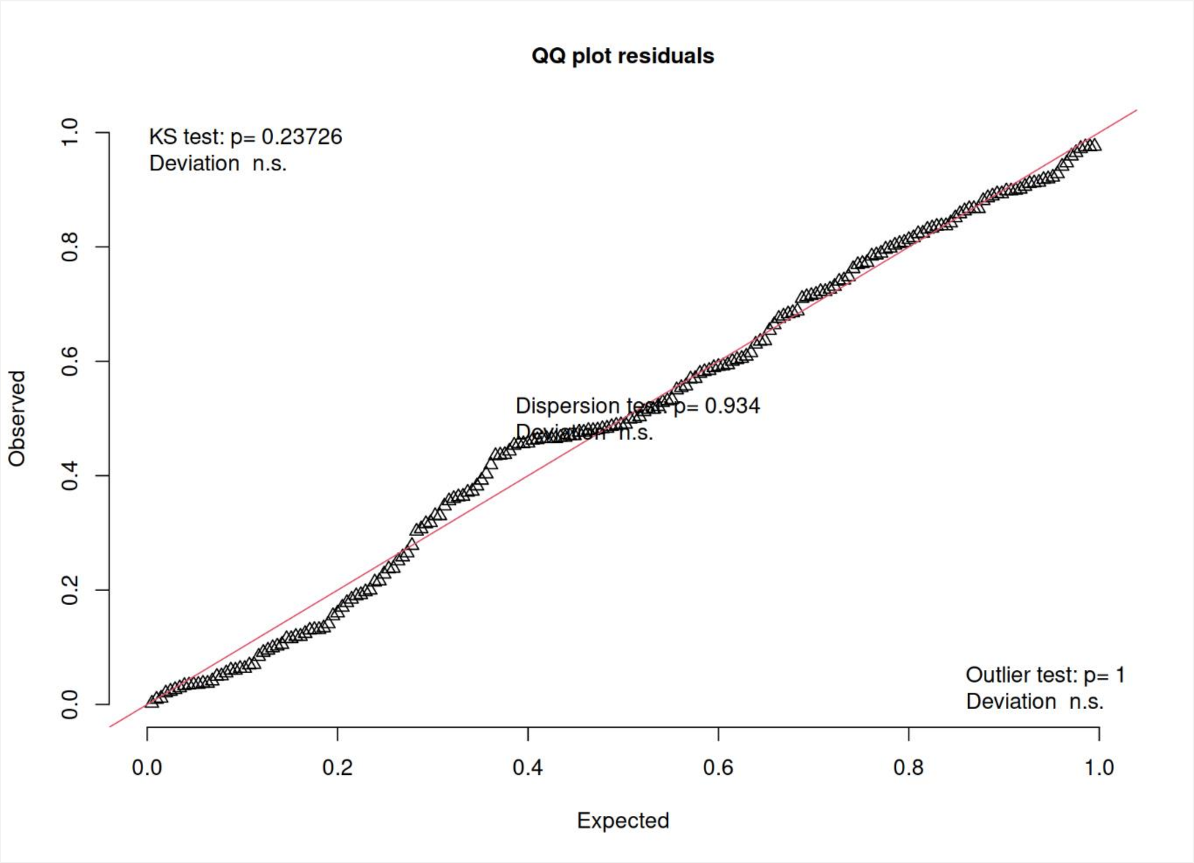

Supplementary Figure 6. DHARMa QQ plot for the Gamma(log) mixed-effects model of tomato percentage reduction in *Phytophthora infestans*-induced late blight severity in tomato plants. Scaled residuals were simulated (n = 1000) from a glmmTMB model fitted to plant-level mean diseased leaf area with treatment as a fixed effect and experimental repetition as a random intercept; a small constant was added to handle zero values. The QQ plot shows close agreement between observed and expected residual distributions, and DHARMa tests indicated no evidence of residual non-uniformity (KS p = 0.237), over/under-dispersion (p = 0.934), or influential outliers (p = 1.0).

2.2 Supplementary Tables

Supplementary Table 1. Analysis of variance (ANOVA) results for bacterial species richness. The effects of plant condition, microcompartment, and soil origin, as well as their interactions, on bacterial species richness are shown. Significant effects are indicated at $p < 0.05$.

Analysis of Variance<br>Response: (richness)					
	Df	Mean sq.	F value	Pr(>F)	Signif. level
Soil origin	1	6651363	152.1425	1.725e-14	*** 0
Plant condition	1	35534	0.8128	0.373285	
Microcompartment	2	2242521	51.2951	2.899e-11	*** 0
Soil.origin: Plant.condition	1	175450	4.0132	0.052717	* 0
Soil.origin: Microcompartment	2	1775944	40.6227	5.885e-10	*** 0
Plant.condition: Microcompartment	2	332586	7.6075	0.001755	** 0.001
Soil origin: Plant condition: Microcompartment	2	90098	2.0609	0.142104	
Signif. codes: 0 ‘***’ 0.001 ‘**’ 0.01 ‘*’ 0.05 ‘.’ 0.1 ‘ ’ 1					

Supplementary Table 2. Analysis of variance (ANOVA) results for Shannon index. The effects of plant condition, microcompartment, and soil origin, as well as their interactions, on bacterial Shannon index are shown. Significant effects are indicated at $p < 0.05$.

Analysis of Variance<br>Response: (Shannon)					
	Df	Mean sq.	F value	Pr(>F)	Signif. level
Soil origin	1	20.2387	59.5805	3.833e-09	***
Plant condition	1	0.1251	0.3683	0.548	
Microcompartment	2	6.9525	20.4673	1.157e-06	***
Soil origin: Plant.condition	1	0 .6079	1.7895	0.189382	
Soil origin: Microcompartment	2	8.6399	25.4349	1.300e-07	***
Plant.condition: Microcompartment	2	2.0763	6.1125	0.005182	**
Soil origin: Plant.condition: Microcompartment	2	1.2456	3.6669	0.035527	*
Signif. codes: 0 ‘***’ 0.001 ‘**’ 0.01 ‘*’ 0.05 ‘.’ 0.1 ‘ ’ 1					

Supplementary Table 3. Analysis of variance (ANOVA) results for species evenness. The effects of plant condition, microcompartment, and soil origin, as well as their interactions, on bacterial species evenness are shown. Significant effects are indicated at $p < 0.05$.

Analysis of Variance<br>Response: (evenness)					
	Df	Mean sq.	F value	Pr(>F)	Signif. level
Soil origin	1	0.055781	15.3792	0.0003787	***
Plant condition	1	0.001626	0.4482	0.5074456	
Microcompartment	2	0.014134	3.8968	0.0293808	*
Soil origin: Plant condition	1	0.005746	1.5843	0.2162427	
Soil origin: Microcompartment	2	0.051315	14.1478	2.925e-05	***
Plant condition: Microcompartment	2	0.016843	4.6436	0.0160653	*
Soil origin: Plant condition: Microcompartment	2	0.017881	4.9300	0.0128128	*
Signif. codes: 0 ‘***’ 0.001 ‘**’ 0.01 ‘*’ 0.05 ‘.’ 0.1 ‘ ’ 1					

Supplementary Table 4. Analysis of variance (ANOVA) results for Simpson index. The effects of plant condition, microcompartment, and soil origin, as well as their interactions, on bacterial Simpson index are shown. Significant effects are indicated at $p < 0.05$.

Analysis of Variance<br>Response: (Simpson)					
	Df	Mean sq.	F value	Pr(>F)	Signif. level
Soil origin	1	0.00261729	6.8400	0.012941	*
Plant condition	1	0.00029956	0.7829	0.382138	
Microcompartment	2	0.00034659	0.9058	0.413238	
Soil origin: Plant.condition	1	0.00006804	0.1778	0.675775	
Soil origin: Microcompartment	2	0.00307463	8.0352	0.001303	**
Plant condition: Microcompartment	2	0.00155411	4.0615	0.025673	*
Soil origin: Plant condition: Microcompartment	2	0.00154188	4.0295	0.026352	*
Signif. codes: 0 ‘***’ 0.001 ‘**’ 0.01 ‘*’ 0.05 ‘.’ 0.1 ‘ ’ 1					

Supplementary Table 5. Permutation test results from Adonis2 for beta diversity analysis with Bray-Curtis dissimilarity. The model assesses the effect of plant condition, microcompartment, and soil origin, as well as their interactions, on bacterial community composition. Significant effects are shown at $p < 0.05$ based on PERMANOVA (Permutational multivariate analysis of variance)

Permutation test for Adonis under the reduced model<br>Terms added sequentially (first to last)<br>Permutation: free<br>Number of permutations: 9999<br>adonis2(formula = ASVs_rela_dist ~ Plant.condition * Microcompartment * Soil.origin, data = metadat, permutations = 9999, method = "Bray")					
	Df	Sumof Sqs	F	Pr(>F)	Signif. level
Plant condition	1	0.2261	1.1624	0.2551	
Microcompartment	2	2.8213	7.2515	0.0001	*** 0
Soil origin	1	1.8771	9.6494	0.0001	*** 0
Plant condition: Microcompartment	2	0.9343	2.4014	0.0001	*** 0
Plant condition: Soil origin	1	0.5224	2.6855	0.0014	** 0.001
Microcompartment: Soil origin	2	3.0007	7.7128	0.0001	*** 0
Plant condition: Microcompartment: Soil origin	2	0.7202	1.8512	0.0062	** 0.001
Signif. codes: 0 ‘***’ 0.001 ‘**’ 0.01 ‘*’ 0.05 ‘.’ 0.1 ‘ ’ 1					
